# Comparative safety and effectiveness of alendronate versus raloxifene in women with osteoporosis

**DOI:** 10.1038/s41598-020-68037-8

**Published:** 2020-07-06

**Authors:** Yeesuk Kim, Yuxi Tian, Jianxiao Yang, Vojtech Huser, Peng Jin, Christophe G. Lambert, Hojun Park, Seng Chan You, Rae Woong Park, Peter R. Rijnbeek, Mui Van Zandt, Christian Reich, Rohit Vashisht, Yonghui Wu, Jon Duke, George Hripcsak, David Madigan, Nigam H. Shah, Patrick B. Ryan, Martijn J. Schuemie, Marc A. Suchard

**Affiliations:** 1 Department of Orthopaedic Surgery, College of Medicine, Hanyang University, Seoul, 04763 Republic of Korea; 2 Department of Computational Medicine, University of California, Los Angeles, CA 90095 USA; 3 Lister Hill National Center for Biomedical Communications, National Library of Medicine, Bethesda, MD 20894 USA; 4 Department of Biomedical Informatics, Columbia University, New York, NY 10032 USA; 5 Department of Internal Medicine, University of New Mexico Health Sciences Center, Albuquerque, NM 87131 USA; 6 Department of Biomedical Informatics, Ajou University, Suwon, 16499 Republic of Korea; 7 Department of Medical Informatics, Erasmus University Medical Center, 3000 Rotterdam, CA The Netherlands; 8 Real World Insights, IQVIA, Cambridge, MA 02139 USA; 9 Department of Medicine, Stanford University School of Medicine, Stanford, CA 94305 USA; 10 School of Biomedical Informatics, The University of Texas Health Science Center at Houston, Houston, TX 77030 USA; 11 Center for Health Analytics and Informatics, Georgia Tech Research Institute, Atlanta, GA 30332 USA; 12 Medical Informatics Services, NewYork-Presbyterian Hospital, New York, NY 10032 USA; 13 Department of Statistics, Columbia University, New York, NY 10032 USA; 14 Epidemiology Analytics, Janssen Research & Development, Titusville, NJ 08560 USA; 15 Department of Biostatistics, Fielding School of Public Health, University of California, Los Angeles, CA 90095 USA; 16 Department of Human Genetics, University of California, Los Angeles, CA 90095 USA

**Keywords:** Computational biology and bioinformatics, Endocrinology, Medical research, Rheumatology

## Abstract

Alendronate and raloxifene are among the most popular anti-osteoporosis medications. However, there is a lack of head-to-head comparative effectiveness studies comparing the two treatments. We conducted a retrospective large-scale multicenter study encompassing over 300 million patients across nine databases encoded in the Observational Medical Outcomes Partnership (OMOP) Common Data Model (CDM). The primary outcome was the incidence of osteoporotic hip fracture, while secondary outcomes were vertebral fracture, atypical femoral fracture (AFF), osteonecrosis of the jaw (ONJ), and esophageal cancer. We used propensity score trimming and stratification based on an expansive propensity score model with all pre-treatment patient characteritistcs. We accounted for unmeasured confounding using negative control outcomes to estimate and adjust for residual systematic bias in each data source. We identified 283,586 alendronate patients and 40,463 raloxifene patients. There were 7.48 hip fracture, 8.18 vertebral fracture, 1.14 AFF, 0.21 esophageal cancer and 0.09 ONJ events per 1,000 person-years in the alendronate cohort and 6.62, 7.36, 0.69, 0.22 and 0.06 events per 1,000 person-years, respectively, in the raloxifene cohort. Alendronate and raloxifene have a similar hip fracture risk (hazard ratio [HR] 1.03, 95% confidence interval [CI] 0.94–1.13), but alendronate users are more likely to have vertebral fractures (HR 1.07, 95% CI 1.01–1.14). Alendronate has higher risk for AFF (HR 1.51, 95% CI 1.23–1.84) but similar risk for esophageal cancer (HR 0.95, 95% CI 0.53–1.70), and ONJ (HR 1.62, 95% CI 0.78–3.34). We demonstrated substantial control of measured confounding by propensity score adjustment, and minimal residual systematic bias through negative control experiments, lending credibility to our effect estimates. Raloxifene is as effective as alendronate and may remain an option in the prevention of osteoporotic fracture.

## Introduction

Osteoporosis is a chronic, progressive disorder characterized by unbalanced bone resorption, decreased bone mass, and deterioration of the bone microarchitecture, leading to decreased bone strength and increased fracture susceptibility^1,2^. Osteoporosis has substantial disease burden worldwide, and postmenopausal women are especially at risk, with prevalence ranging from approximately 20% in the United States and the European Union to nearly 40% in South Korea and Japan^3–5^.

The bisphosphonate alendronate and the selective estrogen receptor modulator (SERM) raloxifene are among the most popular antiresorptive agents for the prevention and treatment of postmenopausal osteoporosis^6,7^. Based on existing randomized studies that compare alendronate and raloxifene separately to placebo^8,9^, alendronate seems to have superior fracture prevention benefits. However, few randomized studies evaluate head-to-head comparative effectiveness of osteoporosis drugs that should inform patient treatment decisions^10^. Observational studies can provide evidence missing from the randomized study literature, especially regarding rare but serious adverse events that require large study populations to detect. Two existing observational studies performed propensity score (PS) adjusted comparative effectiveness analysis on insurance claims databases and find no difference in both vertebral and nonvertebral fracture rates between alendronate and raloxifene patients^3,11^. However, they did not address suspected serious adverse events such as atypical femoral fractures (AFF), esophageal cancer, and osteonecrosis of the jaw (ONJ).

In this paper, we leveraged the research network of the Observational Health Data Sciences and Informatics (OHDSI) collaborative^12^ to conduct a multicenter retrospective cohort study across nine databases investigating comparative risks of fractures and select adverse events among first-time initiators of alendronate and raloxifene. We implemented a suite of methods to address observational study confounding, including propensity score (PS) adjustment to control for measured confounding and negative control experiments, an emerging observational analytics tool^13^, to quantify and adjust for residual study bias.

## Methods

### Data sources

We conducted a new-user cohort study comparing first-time users of alendronate with new users of raloxifene in nine clinical data sources encoded in the Observational Medical Outcomes Partnership (OMOP) Common Data Model (CDM) version 5 from participating research partners across the OHDSI community^12,14,15^. Three data sources were electronic medical records: University of Texas Cerner Health Facts Database (total of 2.4 million [M] patients), Columbia University Medical Center/NewYork-Presbyterian Hospital (4.5M) and Stanford University Hospital (2M). Six data sources are claims records: OptumInsight’s Clinformatics Datamart (Eden Prairie, MN) (CEDM, 40.7M), Truven MarketScan Commercial Claims and Encounters (CCAE, 122M), Truven MarketScan Multi-State Medicaid (MDCD, 17.3M), Truven MarketScan Medicare Supplemental Beneficiaries (MDCR, 9.3M), IQVIA PharMetrics Plus (P-Plus, 105M), and the Korean National Health Insurance Service - National Sample Cohort (NHIS NSC, 1.1M). All were mapped to the Observational Medical Outcomes Partnership Common Data Model (OMOP CDM) schema, providing a homogeneous format for healthcare data and standardization of underlying clinical coding systems that thus enables analysis code to be shared across participating datasets in the network^16,17^. OHDSI network studies are carried out through a federated model, where the access to data and statistical testing executes inside the firewall of the research partners’ infrastructure on de-identified patient information, and the research coordinators collect aggregate results absent of patient-level information for meta-analysis, interpretation, and manuscript generation. Each data partner consulted on a shared study design, including all decisions on cohort definitions and statistical methodology, and presentation of results. Afterwards, each data partner executed an identical study package, so there are no differences in study design across databases.

### Study design

This study followed a retrospective, observational, comparative cohort design^18^. We included women over 45 years old who were first time users of alendronate or raloxifene from January 2001 to February 2012, and who had a diagnosis of osteoporosis in the year prior to treatment initiation. Patients were required to have continuous observation in the database for at least one year prior to treatment initiation and 90 days after. We excluded patients with a previous diagnosis of hip fracture, high-energy trauma, or other diseases related to pathological fractures (including Paget’s disease), as well as patients with prior hip replacements or exposure to any bisphosphonate (including alendronate) or the SERMs raloxifene and bazedoxifene. We used raloxifene as the reference treatment. Full cohort details, including concept codes, are provided in the eMethods in the Supplementary materials.

The primary outcome of interest was osteoporotic hip fracture, while secondary outcomes included vertebral fracture and suspected adverse events: atypical femoral fracture (AFF), osteonecrosis of the jaw (ONJ), and esophageal cancer. We began the outcome risk window at 90 days after treatment initiation, and excluded patients with prior occurrence of that outcome before the risk window. As our primary analysis, we have elected before executing the study to end the outcome time-at-risk window when the patient was no longer observable in the database, analogous to an intent-to-treat design. In addition, to assess the sensitivity of our results to this decision, we considered an alternative analysis in which we ended the time-at-risk window at first cessation of the continuous drug exposure, analogous to an on-treatment design. Continuous drug exposures were constructed from the available longitudinal data by considering sequential prescriptions that had fewer than 30 days gap between prescriptions.

### Ethical considerations

The study was conducted in accordance with the rules of the Declaration of Helsinki of 1975, revised in 2013. The use of Optum and Truven Marketscan databases was reviewed by the New England IRB and was determined to be exempt from broad IRB approval, as this research project did not involve human subjects research. This study was approved with waiver of informed consent by the Columbia University Institutional Review Board under protocol IRB-AAAO7805, most recently renewed 6/11/2019. The research at Stanford was reviewed by their Administrative Panels for the Protection of Human Subjects under protocols 24883 to obtain de-identified data and 53248 to participate in OHDSI network studies. The IRB of Ajou University, Republic of Korea approved the research(AJIRB-MED-EXP-17-24).

### Statistical analysis

We conducted our cohort study using the open-source OHDSI CohortMethod R package^19^, with large-scale analytics achieved through the Cyclops R package^20^. We used propensity scores (PSs)—estimates of treatment exposure probability conditional on pre-treatment baseline features in the 1 year prior to treatment initiation—to control for potential confounding and improve balance between the target (alendronate) and reference (raloxifene) cohorts^21^. We used an expansive PS model that includes all available patient demographic, drug, condition, and procedure covariates instead of a prespecified set of investigator-selected confounders^22^. We performed PS trimming and stratification and then estimated comparative alendronate-vs-raloxifene hazard ratios (HR) using a Cox proportional hazards model. Detailed covariate and methods information are provided in the eMethods in the Supplementary material. We presented PS and covariate balance metrics to assess successful confounding control, and provided hazard ratio estimates and Kaplan–Meier survival plots for the outcomes of interest.

Residual study bias from unmeasured and systematic sources can exist in observational studies after controlling for measured confounding. To estimate such residual bias, we conducted negative control outcome experiments with 147 negative control outcomes^23^, identified through a data-rich algorithm^24^. Negative control outcomes, separate of our study outcomes, are events believed to be unaffected by the studied treatments, thus having a presumed true HR of 1 (See the eMethods in the Supplementary materials for the list of included negative controls). We fitted the negative control estimates to an empirical null distribution that characterizes the study residual bias and is an important artifact from which to assess the study design^25^.

## Results

### Population characteristics

Across all data sources, we identifed 283,586 alendronate patients and 40,463 raloxifene patients for the primary hip fracture analysis, totaling 1,076,597 and 156,080 patient-years of observation, respectively; corresponding cohort sizes for all study outcomes were similar (Table 1). Approximately 98% of patients came from claims databases, and two Electronic Health Records(EHRs)—Columbia and Stanford—had very low numbers of raloxifene users and contributed only modest information (eTable 1 in Supplementary material). The data sources showed a diversity of study entry year and age at study entry distributions (eFigure 1 in Supplementary material). The on-treatment alternative analysis yielded similar cohort sizes for included data sources (eTable 2 in Supplementary material). However, we excluded three data sources (P-Plus, Cerner UT, NHIS NSC) because of continuous drug era encoding difficulties.

**Table 1:** Size of study cohorts for each outcome of interest in primary and alternative analyses.

Outcome	Alendronate				Raloxifene			
	Patients	Person-Years	Events	Rate^a^	Patients	Person-Years	Events	Rate^a^
**Primary analysis**								
Hip fracture	283,586	1,076,597	8051	7.48	40,463	156,080	1033	6.62
Vertebral fracture	279,497	1,058,734	8659	8.18	40,051	154,031	1134	7.36
Atypical femoral fracture	283,894	1,094,049	1244	1.14	40,503	158,722	109	0.69
Esophageal cancer	283,981	1,096,983	234	0.21	40,482	158,858	35	0.22
Osteonecrosis of jaw	284,079	1,097,499	101	0.09	40,511	158,972	9	0.06
**Alternative analysis**^b^								
Hip fracture	185,021	116,262	622	5.35	27,620	17,282	92	5.32
Vertebral fracture	182,025	114,510	719	6.28	27,308	17,066	112	6.56
Atypical femoral fracture	185,258	116,735	85	0.73	27,642	17,345	6	0.35
Esophageal cancer	185,312	116,801	13	0.11	27,625	17,348	$<6$	0.23
Osteonecrosis of jaw	185,367	116,838	$<6$	0.03	27,649	17,365	0	0

^a^Rate: incidence per 1,000 person-years.

^b^Three data sources excluded from alternative analysis (P-Plus, NHIS NSC, Cerner UT).

### Primary outcome assessment

**Fig. 1:**
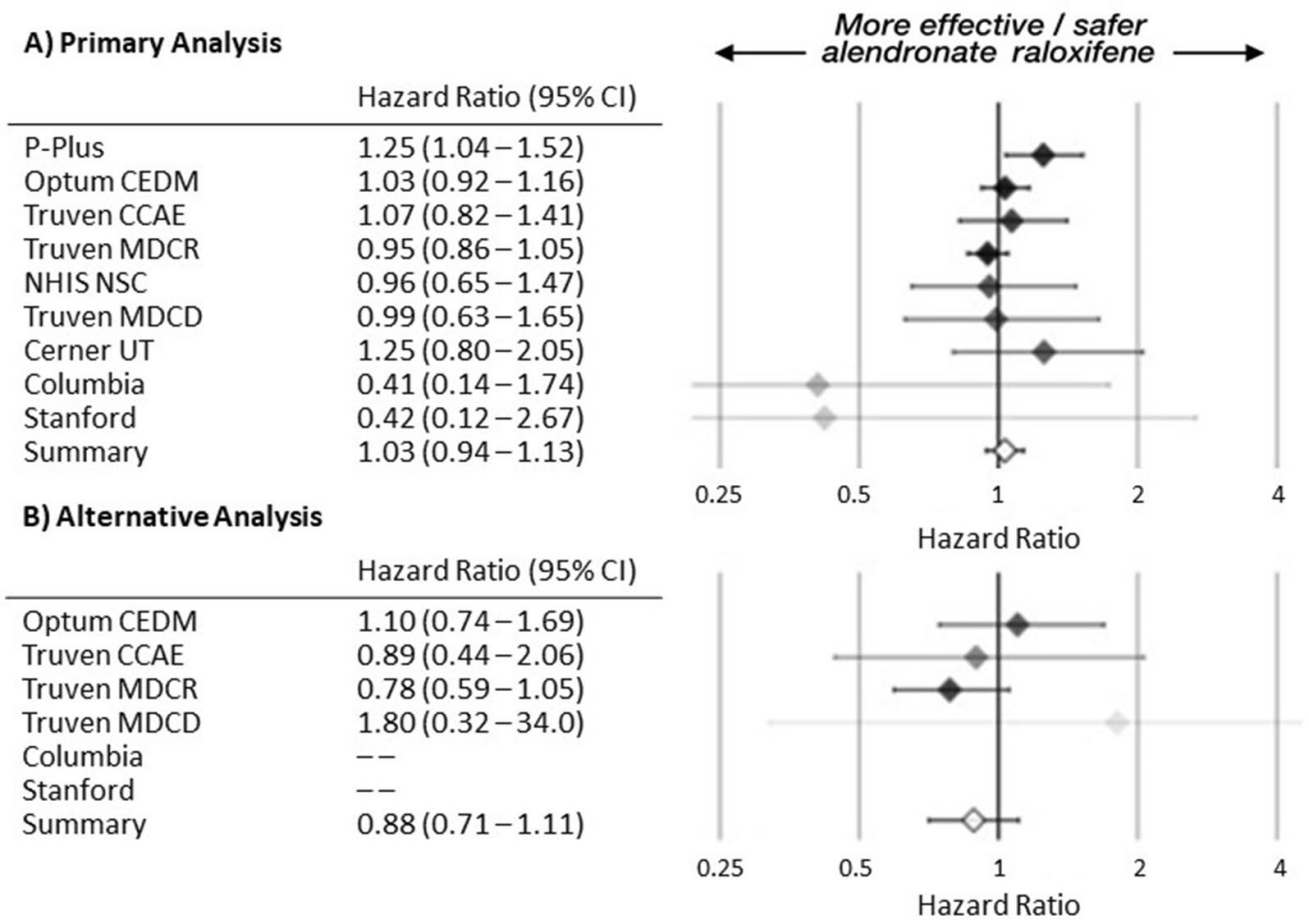
(**A**) Primary and (**B**) alternative analysis hazard ratios (HRs) for hip fracture. More precise estimates have greater opacity. Missing HR from data source with 0 raloxifene events.

In the primary analysis, there were 8,051 hip fractures out of 283,586 patients and the unadjusted rate was 7.48 hip fracture per 1,000 person-years in alendronate. In the raloxifene group, 1,033 out of 40,463 patients had hip fractures with an unadjusted rate of 6.62 hip fractures per 1,000 person-years. The rates in the on-treatment alternative analysis were 5.35 for alendronate and 5.32 for raloxifene (Table 1). Neither the primary analysis across all data sources (summary HR 1.03, 95% CI 0.94–1.13) (Fig. 1a) nor the on-treatment alternative (summary HR 0.88, 95% CI 0.71–1.11) (Fig. 1b) demonstrated a statistically significant difference between treatments. Kaplan-Meier plots across data sources showed small differences between alendronate and raloxifene survival (eFigure 2 in Supplementary material).

### Secondary outcome assessment

**Fig. 2:**
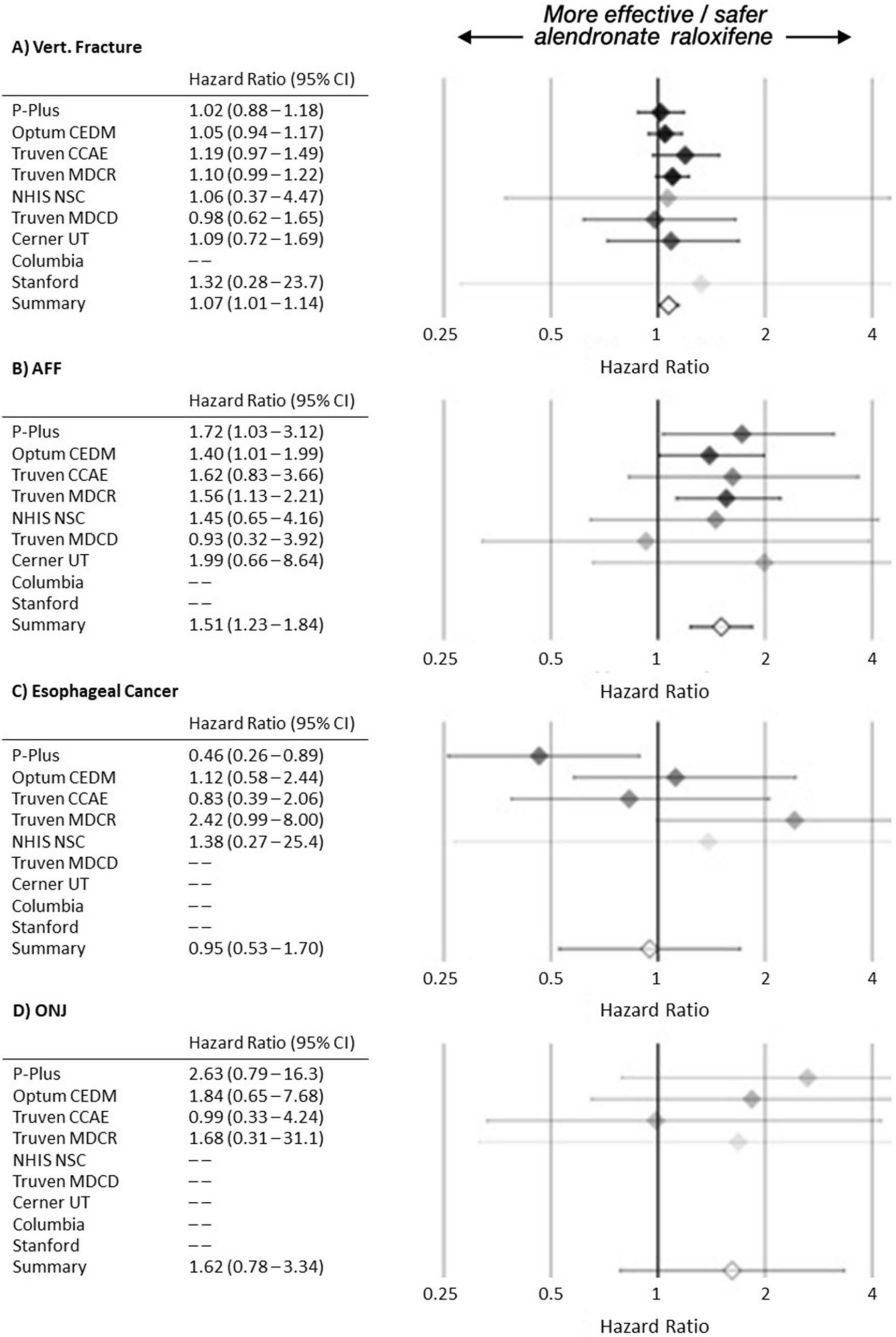
Primary analysis hazard ratios for (**A**) vertebral fracture (Vert. Fracture), (**B**) atypical femoral fracture (AFF), (**C**) esophageal cancer (Eso. Cancer), and (**D**) osteonecrosis of jaw (ONJ). More precise estimates have greater opacity. Missing HR from data sources with 0 raloxifene events.

In the primary analysis, there were 8.18 vertebral fracture, 1.14 AFF, 0.21 esophageal cancer, and 0.09 ONJ outcomes per 1,000 person-years in the alendronate cohort, compared to 7.36, 0.69, 0.22 and 0.06, respectively, in the raloxifene cohort (Table 1). Alendronate users are more likely to have vertebral fractures (summary HR 1.07, 95% CI 1.01–1.14) (Fig. 2a), and have higher risk for AFF (summary HR 1.51, 95% CI 1.23–1.84) (Fig. 2b). There was no significant difference in esophageal cancer risk (summary HR 0.95, 95% CI 0.53–1.70) (Fig. 2c), or ONJ risk (summary HR 1.62, 95% CI 0.78–3.34) (Fig. 2d).

In the on-treatment alternative analysis, the respective rates for the four secondary outcomes were 6.28, 0.73, 0.11, 0.03 among alendronate users and 6.56, 0.35, 0.23, 0.00 among raloxifene users (Table 1). Some data sources had 0 events among one or both treatment groups, and consequently had nonexistent HR estimates. We found no significant vertebral fracture risk (summary HR 0.87, 95% CI 0.71–1.07) and losed power in the other three hypotheses, with extremely wide confidence intervals for AFF and esophageal cancer and 0 raloxifene cohort outcomes for ONJ (eFigure 3 in Supplementary material).

### Cohort balance

Across all data sources, preference score distributions, re-scalings of PS estimates to adjust for differential treatment prevalences, were generally similar and have large overlap between treatment groups, suggesting a meaningful comparative effectiveness study (eFigure 4 in Supplementary material). A large majority of patients had intermediate preference scores, and all data sources except Cerner UT and NHIS NSC displayed at most 10% loss to preference trimming to 0.25–0.75 (Table 2).

**Table 2:** Percentage of cohort eliminated by trimming to 0.25–0.75 preference score.

Data source	Alendronate (%)	Raloxifene (%)	Total (%)
P-Plus	10	11	10
Optum CEDM	7.20	5.90	7
Truven CCAE	3.40	3.80	3.40
Truven MDCR	5.80	6.50	5.90
NHIS NSC	12	17	13
Truven MDCD	7.90	14.00	8.40
Cerner UT	21	19	21
Columbia	0	0	0
Stanford	0	0	0

**Table 3:** Number of covariates by data source, along with mean standardized differences and percentage with standardized difference greater than 0.05 before and after propensity score(PS) adjustment.

Data source	Covariates	Before PS		After PS	
		Mean	$>0.05 (\%)$	Mean	$>0.05 (\%)$
P-Plus	6611	0.23	6.1	0.04	0
Optum CEDM	6890	0.2	8.2	0.05	0.015
Truven CCAE	5605	0.16	4.3	0.05	0
Truven MDCR	4726	0.2	8.8	0.06	0.11
NHIS NSC	3138	0.36	26	0.13	21
Truven MDCD	1873	0.32	53	0.21	49
Cerner UT	721	0.46	72	0.13	20
Columbia	379	0.73	84	0.44	66
Stanford	288	0.45	81	0.44	81

**Fig. 3:**
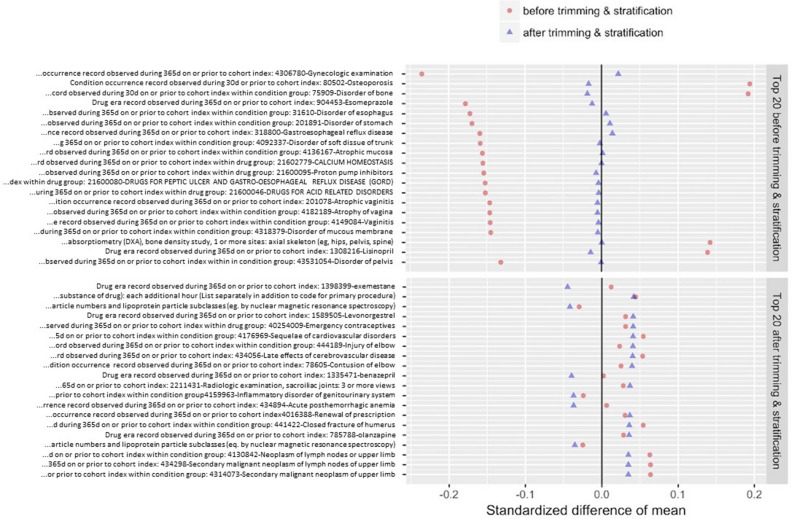
P-Plus: most unbalanced covariates before (top) and after (bottom) PS trimming and stratification.

We assessed the covariate balance achieved through PS adjustment by comparing all covariates’ standardized mean difference between treatment groups before and after PS trimming and stratification, as shown graphically for all data sources (eFigure 5 in Supplementary material), with summary statistics for all data sources shown in Table 3. In all but one data source (Stanford) that had poor PS differentiation, there were large decreases from PS adjustment in both the standardized mean difference and the proportion of covariates with standardized mean difference greater than 0.05. For example, in the P-Plus database, the raloxifene-related covariate “gynecologic examination” is the most unbalanced pre-adjustment covariate, with a standardized mean difference over 0.2. After PS trimming and stratification, this and all other covariates have standardized mean differences smaller than 0.05, indicating successful balancing (Fig. 3).

Considering the top unbalanced covariates in the adjusted cohorts, alendronate users had more bone disorders (eFigures 6–14 in Supplementary material). Meanwhile, raloxifene users had more gastrointestinal counterindications to alendronate and gynecologic examinations and procedures due to its alternate use for breast cancer treatment. These clinical covariates were expected potential confounders and across all data sources became successfully balanced through PS adjustment, reducing the bias in our effect estimates.

### Negative control outcomes

In the absence of bias, 95% of the negative control estimates’ 95% confidence intervals were expected to include the presumed null HR of 1. Across data sources, the proportion of estimates that included 1 was high, ranging from 91 to 96% in primary analysis (eTables 3, 4 in Supplementary material). Furthermore, Gaussian empirical null distributions that estimated the residual bias were centered close to 1 for all data sources except the Columbia and Stanford EHRs that had few raloxifene patients (eTables 5, 6 in Supplementary material). As a result, the theoretical (uncalibrated) and negative control calibrated p-value distributions were very similar to each other (eFigures 15–18 in Supplementary material). These results indicated minimal residual bias across data sources for both primary and alternative analyses, giving further credence to the relative unbiasedness of our treatment effect estimates.

## Discussion

Prevailing clinical wisdom favors alendronate as the first-line treatment option for osteoporosis patients against fracture^26–30^. However, head-to-head randomized studies of alendronate vs raloxifene have only shown increased bone mineral density with alendronate^31,32^, which do not necessarily relate to clinically observed fracture risk^6,9^. Our results found little difference in hip fracture risk between new users of alendronate and raloxifene, and also found a small but statistically significant higher vertebral fracture risk with alendronate. Foster et al. reported non-significantly higher alendronate vertebral fracture risk compared to raloxifene using Truven CCAE and Truven MDCR data^3^. Our data sources were individually similarly non-significant, but together they provided the requisite population size to reveal a statistically significant effect favoring raloxifene.

Growing concern over long-term bisphosphonate use has contributed to steep declines in their prescription^33^. Previous studies report conflicting non-significant^8,34^ and positively significant^35,36^ estimates for atypical femoral fracture risk as a result of bisphosphonate-related suppression of bone remodeling^37^. We found that compared to raloxifene, alendronate did lead to increased AFF risk. Importantly, this well-known and statistically significant risk difference demonstrated that our data sources and study design furnished sufficient statistical power to detect a true difference in the hip fracture HR if one were to exist, given that the rates of AFF were almost an order-of-magnitude less than of hip fracture in our data.

Further, upper gastrointestinal mucosa stimulation is a common bisphosphonate adverse event^38–41^, and concern of bisphosphonate related esophageal cancer has been discussed. Specifically, the US Food and Drug Administration (FDA) received reports of 23 esophageal cancer patients who have taken the alendronate as a suspect drug^42^. After then, the report from the UK primary care cohort concluded that the risk of esophageal cancer increased in patients with oral bisphosphonate compared with non-prescriptions^43^. However, in the following reports, association with the related esophageal cancer is less established^44–46^. We found similar esophageal cancer incidence between alendronate and raloxifene users, and no difference in hazard ratio, and similarly found no difference for osteonecrosis of the jaw, although our study was likely underpowered for this very rare adverse event.

It is known that alendronate related adverse effects such as AFF and ONJ may be affected by the duration of drug use. However, studies to determine duration of use and dosage are lacking. With our study design, we could not establish a dose and duration criterion for analysis. However, our alternative on-treatment analysis was able to measure continuous exposure to treatment, and provide average patient-years of exposure between alendronate and raloxifene cohorts. As calculated from Table 1, for the AFF analysis, alendronate users averaged 0.630 patient-years of continuous exposure, while raloxifene users averaged 0.627 patient-years. For the ONJ analysis, alendronate users averaged 0.630 patient-years of continuous exposure, while raloxifene users averaged 0.628 patient-years of continuous exposure. These are extremely small differences in average exposure duration, giving us confidence in our comparative results for AFF and ONJ.

Many sources of bias unique to retrospective, non-randomized data require attention in order to confidently interpret observational study results. Results may vary from database to database because of differences in study population, and the generalizability of a single study is low^47^. However, due to differences in study implementation, results from different studies often cannot be directly compared. Our study, conducted through the OHDSI community, benefits from a large aggregate study population (over 300,000 patients) and standardized data vocabulary, research protocol, and study implementation. To address confounding due to nonrandom treatment assignment present in all observational studies, we performed PS adjustment using an expansive PS model^22^ that contrasts with the predominant yet inconsistent and potentially biased approach of hand-selecting covariates^48^. We demonstrated substantial improvements in covariate balance from our PS stratification, including balance of covariates related to bone disease severity, alendronate counterindications, and raloxifene’s alternative gynecologic indication.

Meta-analyses often report entirely non-overlapping confidence intervals from different studies investigating the same clinical question. Reported confidence intervals only capture the element of random error, which becomes smaller with larger sample size, but not nonrandom error including study population differences, heterogeneous measurement error, implementation discrepancies, and systematic differences between data sources. Without addressing nonrandom error, divergent study results cannot be reliably combined to leverage the larger aggregate sample sizes across studies. In addition to demonstrating confounding control and using standard research protocols, our study addressed systematic error in each data source through negative control analyses. We use negative controls to quantify systematic bias for this alendronate vs raloxifene comparative effectiveness study, and use the empirical null distribution of negative control estimates to adjust the individual study p-values for our actual outcomes of interest. In this study, we found minimal systematic bias across data sources, providing credibility to our meta-analysis summary hazard ratio estimates.

Our study carries several limitations. Bias from measured and unmeasured sources cannot be ruled out of any observational study, this one included. Data derived from electronic medical records and insurance claims are naturally noisy with missing and misclassified values, and unknown patient histories prior to database entry; our negative control experiments are just one approach to address systematic study bias. Additionally, several of our insurance claims data sources provide much larger study populations that proportionately dominate the smaller data sources in the meta-analysis. As electronic medical records differ in fundamental ways from claims databases, either separate analyses or more complex meta-analysis weighting schemes may accentuate their unique differences. Having said that, several of our participating electronic medical record data sources have very little treatment or outcome data, and may not be as suitable for comparative effectiveness studies. Usually, alendronate has a strong anti-osteoporotic effect and is the preferred treatment for patients with severe osteoporosis. Unfortunately, the severity of osteoporosis as measured through, for example, low bone mineral density scores, was difficult to assess in claim data alone. However, when reviewing unbalanced covariates before and after PS trimming and stratification in our three electric medical records databases (eFigures 12–14 in Supplementary material), we did not find unbalanced covariates representing severe osteoporotic status. We believe it is unlikely that we successfully balance all measured confounders without having balanced osteoporosis severity, despite the lack of mineral density scores such as the T score in our data. Finally, thromboembolism is a concerning adverse effect of raloxifene. The safety of raloxifene in the treatment of osteoporosis was assessed in a large (7,705 patients) multinational, placebo-controlled trial, and during an average of study-drug exposure of 2.6 years, thromboembolism occurred in about 1 out of 100 patients^9^. Recent 2017 guidelines from the American College of Physicians assert that raloxifene should not be used in osteoporotic women due to cerebrovascular and thromboembolic event concerns^49^. With such concern, raloxifene remains a poor treatment choice for patients with high probability of thromboembolic crises. These guidelines are likely to lead to significant channeling bias, in which patients with a high probability of crisis are very unlikely to receive raloxifene treatment, spuriously inflating the unadjusted rate of thromboembolic events among alendronate users. Such strong bias remains difficult to adjust for using PS modeling alone, as there could be little overlap in probability of treatment across high-risk patients. As a consequence, we are unable to report on the relative hazards of thromboembolic events directly.

In our retrospective, head-to-head comparative effectiveness study across nine data sources with common data model, we found that raloxifene was as effective as alendronate, and raloxifene may remain an option in prevention of osteoporotic fracture.

## Supplementary information

Supplementary Information 1

## Data Availability

The data that support the findings of this study are available from the OHDSI study, but restrictions apply to their availability. These data were used under license for the current study and so are not publicly available. The outcome data and codes are, however, available from the authors upon reasonable request and with permission of the OHDSI study.
