# Supplementary material for: Comparative safety and effectiveness of alendronate versus raloxifene in women with osteoporosis
Source: Sci Rep. 2020 Jul 6;10:11115. doi: 10.1038/s41598-020-68037-8 (PMC7338498; doi:10.1038/s41598-020-68037-8)

Supplementary Material

Title: Comparative safety and effectiveness of alendronate versus raloxifene in women with osteoporosis

Yeesuk Kim[1], Yuxi Tian[2], Jianxiao Yang[2], Vojtech Huser[3], Peng Jin[4], Christophe G.Lambert[5], Hojun Park[6], Seng Chan You[6], Rae Woong Park[6], Peter R. Rijnbeek[7], Mui VanZandt[8], Christian Reich[8], Rohit Vashisht[9], Yonghui Wu[10], Jon Duke[11], George Hripcsak[4, 12], David Madigan[13], Nigam H. Shah[9], Patrick B. Ryan[14], Martijn J. Schuemie[14],and Marc A. Suchard[2,15,16]

[1]Department of Orthopaedic Surgery, College of Medicine, Hanyang University, Seoul 04763, Republic of Korea
[2]Department of Computational Medicine, University of California, Los Angeles, CA 90095, USA
[3]Lister Hill National Center for Biomedical Communications, National Library of Medicine, Bethesda, MD 20894,USA
[4]Department of Biomedical Informatics, Columbia University, New York, NY 10032, USA
[5]Department of Internal Medicine, University of New Mexico Health Sciences Center, Albuquerque, NM 87131, USA
[6]Department of Biomedical Informatics, Ajou University, Suwon 16499, Republic of Korea
[7]Department of Medical Informatics, Erasmus University Medical Center, Rotterdam 3000, the Netherlands
[8]Real World Insights, IQVIA, Cambridge, MA 02139, USA
[9]Department of Medicine, Stanford University School of Medicine, Stanford, CA 94305, USA
[10]School of Biomedical Informatics, The University of Texas Health Science Center at Houston, TX 77030, USA
[11]Center for Health Analytics and Informatics, Georgia Tech Research Institute, Atlanta, GA 30332, USA
[12]Medical Informatics Services, NewYork-Presbyterian Hospital, New York, NY 10032, USA
[13]Department of Statistics, Columbia University, New York, NY 10032, USA
[14]Epidemiology Analytics, Janssen Research & Development, Titusville, NJ 08560, USA
[15]Department of Biostatistics, Fielding School of Public Health, University of California, Los Angeles, CA 90095,USA
[16]Department of Human Genetics, University of California, Los Angeles, CA 90095, USA

1. eMethods
1) Cohort Definition
2) Outcome Definitions
3) Covariate Sets
4) Negative controls
5) Statistical analysis

2. eTables
eTable 1. Number of patients, observation years, and number of events in study cohort by data source in primary analysis
eTable 2. Number of patients, observation years, and number of events in study cohort by data source in Alternative analysis
eTable 3. Empirical null distribution constructed from negative controls, and the number of estimates that do not reject the null effect hypothesis. Primary analysis
eTable 4. Empirical null distribution constructed from negative controls, and the number of estimates that do not reject the null effect hypothesis. Alternative analysis
eTable 5. Original estimates and p-values for hip fracture primary analysis, with negative control calibrated p-values.
eTable 6. Original estimates and p-values for hip fracture alternative analysis, with negative control calibrated p-values.

3. eFigures
eFigure 1. A) year of and B) age at study entry, stratified by drug exposure and data source. Note patient counts are on the log-scale
eFigure 2. Kaplan-Meier curves for Hip Fracture outcome
eFigure 3. Outcome Assessments of Alternative Analysis in vertebral fracture and adverse events
eFigure 4. Preference Score distributions in original cohort (left) and after trimming and stratification (right)
eFigure 5. Standardized difference of covariate (1 dot = 1 covariate) in each study population before and after propensity score trimming and stratification
eFigure 6. P-Plus: most unbalanced covariates before (top) and after (bottom) PS trimming and stratification
eFigure 7. Optum CEDM: most unbalanced covariates before (top) and after (bottom) PS trimming and stratification
eFigure 8. Truven CCAE: most unbalanced covariates before (top) and after (bottom) PS trimming and stratification
eFigure 9. Truven MDCR: most unbalanced covariates before (top) and after (bottom) PS trimming and stratification
eFigure 10. NHIS NSC: most unbalanced covariates before (top) and after (bottom) PS trimming and stratification
eFigure 11. Truven MDCD: most unbalanced covariates before (top) and after (bottom) PS trimming and stratification
eFigure 12. Cerner UT: most unbalanced covariates before (top) and after (bottom) PS trimming and stratification

eFigure 13. Columbia: most unbalanced covariates before (top) and after (bottom) PS trimming and stratification
eFigure 14. Stanford: most unbalanced covariates before (top) and after (bottom) PS trimming and stratification
eFigure 15. Primary analysis negative control estimates with traditional and calibrated significance testing.
eFigure 16. Alternative analysis negative control estimates with traditional and calibrated significance testing.
eFigure 17. Primary analysis p-value calibration plot showing the fraction of negative controls with p < α, for different levels of α.
eFigure 18. Alternative analysis p-value calibration plot showing the fraction of negative controls with p < α, for different levels of α.

eMethods

1) Cohort Definitions

Alendronate new users

Initial Event Cohort

People having any of the following:

• a drug era of alendronate[1]

◦ for the first time in the person's history ◦ occurrence start is before 2012-02-01

with continuous observation of at least 183 days prior and 0 days after event index date, and limit initial events to: earliest event per person.

Inclusion Criteria 1: is woman > 45 yo

Having all of the following criteria:

• with the following event criteria:

◦ with age > 45

◦ gender is any of: FEMALE

Inclusion Criteria 2: has osteoporosis diagnosis in 365d prior to index date

Having all of the following criteria:

• at least 1 occurrences of a condition occurrence of osteoporosis[6] starting between 365 days Before and 0 days After event index date

Inclusion Criteria 3: has no prior raloxifene exposure

Having all of the following criteria:

• exactly 0 occurrences of a drug exposure of raloxifene[7] starting between all days Before and 0 days After event index date

Inclusion Criteria 4: has no prior hip fractures

Having all of the following criteria:

• exactly 0 occurrences of a condition occurrence of hip fracture[4] starting between all days Before and 0 days After event index date

Inclusion Criteria 5: has no prior hip replacement

Having all of the following criteria:

• exactly 0 occurrences of a procedure of Hip replacement[5] starting between all days Before and 0 days After event index date

Inclusion Criteria 6: has no prior drugs that should be excluded

Having all of the following criteria:

• exactly 0 occurrences of a drug exposure of Sisyphus challenge:[8] starting between all days Before and 1 day Before event index date

Inclusion Criteria 7: no prior disease related to pathological fractures including Pagets disease

Having all of the following criteria:

• exactly 0 occurrences of a condition occurrence of diseases related to pathological fractures[2] starting between all days Before and 0 days After event index date

Inclusion Criteria 8: no prior high energy trauma fractures

Having all of the following criteria:

• exactly 0 occurrences of a condition occurrence of high energy trauma fractures[3] starting between all days Before and 0 days After event index date

Custom Cohort Exit Criteria

This strategy creates a drug era from the codes found in the specified concept set. If the index event is found within an era, the cohort end date will use the era's end date. Otherwise, it will use the observation period end date that contains the index event.

Use the era end date of alendronate[1]

• allowing 30 days between exposures

• adding 0 days after exposure end

Raloxifene new users

Initial Event Cohort

People having any of the following:

• a drug era of raloxifene[7]

  ◦ for the first time in the person's history

  ◦ occurrence start is before 2012-02-01

with continuous observation of at least 183 days prior and 0 days after event index date, and limit initial events to: earliest event per person.

Inclusion Criteria 1,2, 4-8: Same as Alendronate Cohort

Inclusion Criteria 3: has no prior alendronate exposure

Having all of the following criteria:

• exactly 0 occurrences of a drug exposure of alendronate[1] starting between all days Before and 0 days After event index date

Custom Cohort Exit Criteria

Use the era end date of raloxifene[7]

• allowing 30 days between exposures

• adding 0 days after exposure end

Appendix: Concept Set Definitions

Codes given use the Observational Medical Outcomes Partnership Common Data Model Version 5 format. The "Vocabulary" column indicates the source vocabulary set for the concept. The "Excluded" column indicates whether the covariate is included (NO) or excluded (YES) from the cohort definition. The "Descendants" column incidates whether all descendent concepts in the vocabulary hierarchy are also incorporated.

1. alendronate

Concept Id	Concept Name	Domain	Vocabulary	Excluded	Descendants
1557272	alendronate	Drug	RxNorm	NO	YES

2. diseases related to pathological fractures

Concept Id	Concept Name	Domain	Vocabulary	Excluded	Descendants
444204	Neoplasm of bone	Condition	SNOMED	NO	YES
75910	Osteitis deformans	Condition	SNOMED	NO	YES
73571	Pathological fracture	Condition	SNOMED	NO	YES

3. high energy trauma fractures

Concept Id	Concept Name	Domain	Vocabulary	Excluded	Descendants
4300192	Fracture of pelvis	Condition	SNOMED	NO	YES
4264281	Open fracture	Condition	SNOMED	NO	YES

4. hip fracture

Concept Id	Concept Name	Domain	Vocabulary	Excluded	Descendants
4230399	Closed fracture of hip	Condition	SNOMED	NO	YES

5. Hip replacement

Concept Id	Concept Name	Domain	Vocabulary	Excluded	Descendants

2104836	Arthroplasty, acetabular and proximal femoral prosthetic replacement (total hip arthroplasty), with or without autograft or allograft)	Procedure	CPT4	NO	YES
4142076	Primary cemented total hip replacement	Procedure	SNOMED	NO	YES
4079259	Primary uncemented total hip replacement	Procedure	SNOMED	NO	YES
2005891	Total hip replacement	Procedure	ICD9Proc	NO	YES
4001859	Hip joint implantation	Procedure	SNOMED	NO	YES
4162099	Prosthetic arthroplasty of the hip	Procedure	SNOMED	NO	YES
4010119	Revision of hip replacement	Procedure	SNOMED	NO	YES
4203771	Total replacement of hip	Procedure	SNOMED	NO	YES
4207955	Insertion of hip prosthesis, total	Procedure	SNOMED	NO	YES
4297365	Partial hip replacement by prosthesis	Procedure	SNOMED	NO	YES
2005902	Partial hip replacement	Procedure	ICD9Proc	NO	YES
4266062	Revision of total hip replacement	Procedure	SNOMED	NO	YES
4197231	Removal of prosthesis of joint structures of hip	Procedure	SNOMED	NO	YES

6. osteoporosis

Concept Id	Concept Name	Domain	Vocabulary	Excluded	Descendants
80502	Osteoporosis	Condition	SNOMED	NO	YES

7. raloxifene

Concept Id	Concept Name	Domain	Vocabulary	Excluded	Descendants
1513103	Raloxifene	Drug	RxNorm	NO	YES

8. Sisyphus challenge: drugs to exclude

Concept Id	Concept Name	Domain	Vocabulary	Excluded	Descendants
1557272	Alendronate	Drug	RxNorm	NO	YES
44506794	bazedoxifene	Drug	RxNorm	NO	YES
21604148	Bisphosphonates	Drug	ATC	NO	YES
1513103	Raloxifene	Drug	RxNorm	NO	YES

2) Outcome Definitions

Hip Fracture New Events

Initial Event Cohort

People having any of the following:

• a condition occurrence of hip fracture[1]

◦ for the first time in the person’s history

with continuous observation of at least 0 days prior and 0 days after event index date, and limit initial events to: earliest event per person.

Vertebral Fracture New Events

Initial Event Cohort

People having any of the following:

• a condition occurrence of closed vertebral fracture[2]

◦ for the first time in the person’s history

with continuous observation of at least 0 days prior and 0 days after event index date, and limit initial events to: earliest event per person.

Custom Cohort Exit Criteria

This strategy creates an era from the codes found in the specified concept set. If the index event is found within an era, the cohort end date will use the era’s end date. Otherwise, it will use the observation period end date that contains the index event.

Use the era end date of closed vertebral fracture[2]

• allowing 30 days between exposures

• adding 0 days after exposure end

Atypical Femoral Fracture New Events

Initial Event Cohort

People having any of the following:

• a condition occurrence of atypical femural fractures[3]

◦ for the first time in the person’s history

with continuous observation of at least 0 days prior and 0 days after event index date, and limit initial events to: earliest event per person.

Inclusion Criteria 1: no high energy trauma fractures around atypical fracture event

Having all of the following criteria:

• exactly 0 occurrences of a condition occurrence of high energy trauma fractures[4] starting between 7 days Before and 7 days After event index date

Esophageal Cancer New Events

Initial Event Cohort

People having any of the following:

• a condition occurrence of esophageal cancer[5]

◦ for the first time in the person’s history

with continuous observation of at least 0 days prior and 0 days after event index date, and limit initial events to: earliest event per person.

Osteonecrosis of Jaw New Events

Initial Event Cohort

People having any of the following:

• a condition occurrence of Osteonecrosis of jaw[6]

◦ for the first time in the person’s history

with continuous observation of at least 0 days prior and 0 days after event index date, and limit initial events to: earliest event per person.

Appendix: Concept Set Definitions

1. hip fracture

Concept Id	Concept Name	Domain	Vocabulary	Excluded	Descendants
4230399	Closed fracture of hip	Condition	SNOMED	NO	YES

2. closed vertebral fracture

Concept Id	Concept Name	Domain	Vocabulary	Excluded	Descendants
4170742	Closed fracture of vertebral column	Condition	SNOMED	NO	YES

3. atypical femural fractures

Concept Id	Concept Name	Domain	Vocabulary	Excluded	Descendants
4009610	Closed fracture proximal femur, subtrochanteric	Condition	SNOMED	NO	YES
4264281	Open fracture	Condition	SNOMED	NO	YES

4. high energy trauma fractures

Concept Id	Concept Name	Domain	Vocabulary	Excluded	Descendants
4300192	Fracture of pelvis	Condition	SNOMED	NO	YES
4264281	Open fracture	Condition	SNOMED	NO	YES

5. esophageal cancer

Concept Id	Concept Name	Domain	Vocabulary	Excluded	Descendants
4181343	Malignant tumor of esophagus	Condition	SNOMED	NO	YES

6. Osteonecrosis of jaw

Concept Id	Concept Name	Domain	Vocabulary	Excluded	Descendants
40480852	Aseptic necrosis of bone of jaw	Condition	SNOMED	NO	YES
46270478	Osteonecrosis of jaw caused by drug	Condition	SNOMED	NO	YES

3) Covariate Sets

- Demographics (age in 5-year increments, gender, year of index date, month of index date)
- Condition Occurrence (condition occurrence in lookback window)
    - in 365 days prior to index date
    - in 30 days prior to index date
- Condition Group (total SNOMED condition record count)
- Drug Era (span of time when person assumed to have drug)
    - in 365 days prior to index date
- Drug Group (total drug record count)
- Procedure Occurrence
    - in 365 days prior to index date
- Measurement
    - in 365 days prior to index date
    - measurements count in 365 days prior to index date
- Concepts Count in 365 days prior to index date
- Risk Scores (Charlson)

4) Negative controls

Negative controls were selected using the following criteria, as detailed in Voss et al[1].

• No evidence found in literature on clinical trials using the method proposed by Avillach et al[2].

• No evidence found in literature using the method used in SemMedDB[3]

• No evidence found in the structured product label (US and EU).

• FAERS Proportional Reporting Ratio (PRR) needed to be less than 2.

Edema of larynx	Meningitis	Neoplasm of tongue
Restless legs	Hypovolemia	Acute skin disorder
Irritable bowel syndrome	Disease due to Arthropod	Chronic heart disease
Rheumatoid arthritis	Iron deficiency anemia	Anorectal disorder
Pyoderma	Intracranial injury	Injury of eye region
Pityriasis versicolor	Streptococcal infectious disease	Encephalomyelopathy
Aplastic anemia	Hypersomnia	Hemochromatosis
Thyrotoxicosis	Mediastinitis	Lower respiratory tract infection
Ingrowing nail	Wound dehiscence	Mass of urinary bladder
Chronic leukemia	Acute stress disorder	Chronic arthropathy
Infectious disorder of kidney	Parasomnia	Bacterial gastrointestinal infectious disease
Cystic disease of kidney	Mental retardation	Viral infection of the digestive tract
Hemorrhoids	Appendicitis	Heart valve stenosis
Urethritis	Mycobacteriosis	Lesion of bronchus
Septic shock	Chronic pain syndrome	Anemia due to intrinsic red cell abnormality
Gallstone	Cystic fibrosis	Thrombosis of vein of trunk
Calculus of lower urinary tract	Personality disorder	Infective otitis media
Vesicoureteric reflux	Burn	Ketoacidosis
Large liver	Oligomenorrhea	Hyperglycemia
Pyelonephritis	Vascular dementia	Acrodermatitis
Occlusion of ureter	Diabetic oculopathy	Bladder dysfunction
Acute cholecystitis	Herpes simplex	Disease due to Gammaherpesvirinae
Injury of abdomen	Traumatic hemorrhage	T-cell AND/OR NK-cell neoplasm
Type 1 diabetes mellitus	Irritant contact dermatitis	Tonsillitis
Bronchopneumonia	Gammopathy	Respiratory failure
Atelectasis	Seventh cranial nerve finding	Glomerulonephritis

Congenital heart disease	Disease due to Paramyxoviridae	Musculoskeletal fibromatosis
Venous hypertension	Folliculitis	Substance abuse
Paroxysmal tachycardia	Upper urinary tract dilatation and obstruction	Thrombocytosis
Aortic valve disorder	Hodgkin's disease	Autoimmune thyroiditis
Cardiomegaly	Non-Hodgkin's lymphoma	Type B viral hepatitis
Lymphadenitis	Generalized epilepsy	Schizoaffective disorder
Peripheral arterial occlusive disease	Inflammatory bowel disease	Gastrointestinal fistula
Secondary hypertension	Superficial mycosis	Phobic disorder
Optic atrophy	Infection of nail	Chronic myeloproliferative disorder
Cerebral ischemia	Animal-induced dermatosis	Abnormal breathing
Demyelinating disease of central nervous system	Corneal endothelium finding	Macrocytic anemia
Amblyopia	Anterior chamber finding	Anemia due to substance
Blepharitis	Urinary tract pain	Pulmonary necrosis
Trigeminal neuralgia	Inguinal canal finding	Strabismus
Keratoconjunctivitis	Finding of bowel continence	Tendon injury
Tic disorder	Lesion of rectum	Nocturia
Symbolic dysfunction	Complete bilateral paralysis	Ventricular septal abnormality
Delusional disorder	Partial seizure	Toxic metabolic encephalopathy
Hemoglobinopathy	Pulmonary valve finding	Paralytic syndrome of all four limbs
Disease due to Retroviridae	Toxic pneumonitis	Paralytic syndrome of both lower limbs
Impulse control disorder	Immune thrombocytopenic purpura	Paralytic syndrome on one side of the body
Spirochetal infection	Hypertrophic cardiomyopathy	Precapillary pulmonary hypertension
Schizophrenia	Neoplasm of pancreas	Acquired brain injury

References

1. Voss EA, Boyce RD, Ryan PB, van der Lei J, Rijnbeek PR, Schuemie MJ. Accuracy of an automated knowledge base for identifying drug adverse reactions. *J Biomed Inform.* 2017;66:72-81.
2. Avillach P, Dufour JC, Diallo G, et al. Design and validation of an automated method to detect known adverse drug reactions in MEDLINE: a contribution from the EU-ADR project. *J Am Med Inform Assoc.* 2013;20(3):446-452.
3. Kilicoglu H, Rosemblat G, Fiszman M, Rindflesch TC. Constructing a semantic predication gold standard from the biomedical literature. *BMC Bioinformatics.* 2011;12:486.

4) Statistical analysis

We estimate propensity scores using a logistic regression model. We include all available pretreatment

patient covariates in the PS model (e.g. all conditions, all procedures) instead of a prespecified set of investigator-selected confounders. We use L1-regularization to conduct a penalized regression, and use 10-fold cross-validation to select the penalty hyperparameter.

We transform propensity scores to preference scores that account for differences in drug utilization, trim the preference scores to 0.25-0.75, then create five equally-sized strata. We use the PS-adjusted strata in a stratified Cox proportional hazards outcome model with treatment exposure as the only covariate. We report 95% confidence intervals obtained from the profile likelihood. We combine estimates from data sources into a summary hazard ratio using a random effects model meta-analysis.

eTable 1. Number of patients, observation years, and number of events in study cohort by data source in primary analysis

	Alendronate			Raloxifene		
Data source	Patients	Years	Events	Patients	Years	Events
			Hip Fracture			
P-Plus	78,155	245,336	1,216	10,742	34,711	117
Optum CEDM	67,100	262,467	2,495	10,167	40,528	323
Truven CCAE	64,003	228,085	432	10,534	38,655	63
Truven MDCR	47,576	210,908	3,247	6,459	29,840	457
NHIS NSC	17,766	94,139	313	1,314	7,823	26
Truven MDCD	4,570	16,454	209	369	1,340	19
Cerner UT	2,644	8,867	100	787	2,740	23
Columbia	1,131	7,696	24	49	298	<6
Stanford	641	2,645	15	42	145	<6
Total	283,586	1,076,597	8,051	40,463	156,080	1,033
			Vertebral Fracture			
P-Plus	77,163	241,612	1,603	10,662	34,246	203
Optum CEDM	65,990	256,492	2,842	10,078	39,852	383
Truven CCAE	63,414	225,516	711	10,461	38,319	98
Truven MDCR	46,416	205,679	3,105	6,328	29,382	398
NHIS NSC	17,766	94,802	31	1,315	7,884	<6
Truven MDCD	4,463	15,970	226	353	1,263	18
Cerner UT	2,543	8,484	102	766	2,643	30
Columbia	1,122	7,654	16	48	304	0
Stanford	620	2,525	23	40	138	<6
Total	279,497	1,058,734	8,659	40,051	154,031	1,134
			Atypical Femoral Fracture			
P-Plus	78,242	247,511	189	10,753	34,973	14
Optum CEDM	67,176	268,136	398	10,178	41,372	38
Truven CCAE	64,031	229,054	81	10,535	38,803	8
Truven MDCR	47,700	218,528	428	6,469	31,081	38
NHIS NSC	17,753	94,587	84	1,316	7,881	<6
Truven MDCD	4,579	16,855	35	369	1,378	<6

Cerner UT	2,641	8,954	20	792	2,782	<6
Columbia	1,132	7,760	6	49	304	0
Stanford	640	2,664	<6	42	148	0
Total	283,894	1,094,049	1,244	40,503	158,722	109
			Esophageal Cancer			
P-Plus	78,253	247,863	46	10,751	34,966	14
Optum CEDM	67,202	269,103	61	10,167	41,430	9
Truven CCAE	64,023	229,173	37	10,528	38,791	7
Truven MDCR	47,735	219,626	68	6,470	31,168	<6
NHIS NSC	17,759	94,787	14	1,316	7,888	<6
Truven MDCD	4,580	16,937	7	369	1,381	0
Cerner UT	2,657	9,052	0	790	2,782	0
Columbia	1,131	7,769	<6	49	304	0
Stanford	641	2,673	0	42	148	0
Total	283,981	1,096,983	234	40,482	158,858	35
			Osteonecrosis of Jaw			
P-Plus	78,277	247,899	34	10,754	35,000	<6
Optum CEDM	67,214	269,170	36	10,181	41,479	<6
Truven CCAE	64,043	229,256	20	10,536	38,810	<6
Truven MDCR	47,750	219,770	10	6,472	31,173	<6
NHIS NSC	17,778	94,921	0	1,316	7,892	0
Truven MDCD	4,586	16,970	<6	369	1,381	0
Cerner UT	2,657	9,052	0	792	2,785	0
Columbia	1,133	7,788	0	49	304	0
Stanford	641	2,673	0	42	148	0
Total	284,079	1,097,499	101	40,511	158,972	9

eTable2. Number of patients, observation years, and number of events in study cohort by data source in Alternative analysis

	Alendronate			Raloxifene		
Data source	Patients	Years	Events	Patients	Years	Events
			Hip Fracture			
Optum CEDM	67,100	37,601	219	10,167	6,055	27
Truven CCAE	64,003	38,103	48	10,534	6,057	8
Truven MDCR	47,576	36,512	340	6,459	4,969	56
Truven MDCD	4,570	1,209	11	369	150	<6
Columbia	1,131	1,995	<6	49	17	0
Stanford	641	842	<6	42	34	0
Total	185,021	116,262	622	27,620	17,282	92
			Vertebral Fracture			
Optum CEDM	65,990	37,021	260	10,078	5,974	43
Truven CCAE	63,414	37,823	90	10,461	6,011	16
Truven MDCR	46,416	35,713	332	6,328	4,893	51
Truven MDCD	4,463	1,164	20	353	137	<6
Columbia	1,122	1,977	6	48	17	0
Stanford	620	812	11	40	34	0
Total	182,025	114,510	719	27,308	17,066	112
			Atypical Femoral Fracture			
Optum CEDM	67,176	37,732	34	10,178	6,072	<6
Truven CCAE	64,031	38,157	8	10,535	6,065	0
Truven MDCR	47,700	36,795	41	6,469	5,007	<6
Truven MDCD	4,579	1,212	<6	369	150	0
Columbia	1,132	1,997	0	49	17	0
Stanford	640	842	0	42	34	0
Total	185,258	116,735	85	27,642	17,345	<6
			Esophageal Cancer			
Optum CEDM	67,202	37,751	<6	10,167	6,068	<6
Truven CCAE	64,023	38,151	<6	10,528	6,059	<6
Truven MDCR	47,735	36,854	6	6,470	5,020	<6

Truven MDCD	4,580	1,212	<6	369	150	0
Columbia	1,131	1,991	0	49	17	0
Stanford	641	842	0	42	34	0
Total	185,312	116,801	13	27,625	17,348	<6
			Osteonecrosis of Jaw			
Optum CEDM	67,214	37,759	<6	10,181	6,078	0
Truven CCAE	64,043	38,163	<6	10,536	6,063	0
Truven MDCR	47,750	36,860	0	6,472	5,023	0
Truven MDCD	4,586	1,215	0	369	150	0
Columbia	1,133	1,999	0	49	17	0
Stanford	641	842	0	42	34	0
Total	185,367	116,838	<6	27,649	17,365	0

eTable 3. Empirical null distribution constructed from negative controls, and the number of estimates that do not reject the null effect hypothesis. Empirical confidence interval(CI) are from the profile likelihood, theoretical p-values are from the likelihood asymptotic distribution and calibrated p-values are from the negative control calibrated standard errors. For the calibrated p-value, a leave-one-out design was used. Results by data source for primary analysis

	Empirical Null Distribution			Coverage of Null Effect		
Data source	Mean	$SD	Controls	Empirical CI	Theoretical p	Calibrated p
P-Plus	-0.00803	0.0352	147	135 (92%)	135 (92%)	138 (94%)
Optum CEDM	-0.0106	0.0157	147	141 (96%)	143 (97%)	142 (97%)
Truven CCAE	-0.0221	0.014	146	139 (95%)	139 (95%)	141 (97%)
Truven MDCR	-0.0345	0.0201	146	133 (91%)	133 (91%)	135 (92%)
NHIS NSC	-0.00491	0.0162	122	117 (96%)	119 (98%)	118 (97%)
Truven MDCD	-0.0462	0.0247	126	120 (95%)	123 (98%)	122 (97%)
Cerner UT	0.0627	0.0373	105	99 (94%)	103 (98%)	102 (97%)
Columbia	-0.542	0.0178	53	51 (96%)	51 (96%)	52 (98%)
Stanford	-0.964	0.0816	53	32 (91%)	32 (91%)	35 (100%)

$SD: standard deviation

eTable 4. Empirical null distribution constructed from negative controls, and the number of estimates that do not reject the null effect hypothesis. Empirical confidence interval(CI) are from the profile likelihood, theoretical p-values are from the likelihood asymptotic distribution and calibrated p-values are from the negative control calibrated standard errors. For the calibrated p-value, a leave-one-out design was used. Results by data source for alternative analysis

	Empirical Null Distribution			Coverage of Null Effect		
Data source	Mean	$SD	Controls	Empirical CI	Theoretical p	Calibrated p
Optum CEDM	-0.119	0.0494	138	124 (90%)	123 (89%)	131 (95%)
Truven CCAE	-0.0801	0.015	128	119 (93%)	119 (93%)	123 (96%)
Truven MDCR	-0.0955	0.0206	130	121 (93%)	121 (93%)	125 (96%)
Truven MDCD	-0.263	0.0388	69	67 (97%)	68 (99%)	67 (97%)
Columbia	-2.4	0.0643	9	7 (78%)	2 (22%)	9 (100%)
Stanford	-1.87	0.0866	20	16 (80%)	15 (75%)	20 (100%)

$SD: standard deviation

eTable 5. Original estimates and p-values for primary analysis, with negative control calibrated p-values. Bounds on calibrated p-values calculated from the 95% bounds of original estimate

	Original Estimate			Calibrated P-value		
Data source	Mean	SD	p-value	p-value	95% ¶lb	95% §ub
			Hip Fracture			
P-Plus	0.223	0.0982	0.0233	0.0316	0.016	0.0731
Optum CEDM	0.0328	0.0601	0.585	0.5	0.358	0.675
Truven CCAE	0.0657	0.137	0.631	0.528	0.441	0.624
Truven MDCR	-0.0543	0.0508	0.286	0.738	0.513	0.959
NHIS NSC	-0.0449	0.209	0.83	0.849	0.748	0.957
Truven MDCD	-0.0125	0.246	0.96	0.887	0.7	0.994
Cerner UT	0.227	0.241	0.346	0.519	0.316	0.765
Columbia	-0.9	0.645	0.163	0.581	0.386	0.798
Stanford	-0.865	0.795	0.276	0.867	0.6	0.994
			Vertebral Fracture			
P-Plus	0.0165	0.0758	0.827	0.779	0.615	0.958
Optum CEDM	0.0458	0.0553	0.408	0.345	0.223	0.516
Truven CCAE	0.176	0.109	0.106	0.0759	0.0499	0.115
Truven MDCR	0.0937	0.0539	0.0822	0.0378	0.0132	0.118
NHIS NSC	0.0611	0.634	0.923	0.917	0.882	0.952
Truven MDCD	-0.0204	0.251	0.935	0.907	0.728	0.995
Cerner UT	0.0841	0.217	0.698	0.881	0.637	0.994
Columbia	NA	NA	NA	NA	NA	NA
Stanford	0.279	1.13	0.805	0.279	0.172	0.427
			Atypical Femoral Fracture			
P-Plus	0.544	0.282	0.0542	0.0535	0.0455	0.0655
Optum CEDM	0.334	0.172	0.053	0.0479	0.0386	0.0607
Truven CCAE	0.483	0.379	0.203	0.184	0.168	0.202
Truven MDCR	0.442	0.172	0.0101	0.00632	0.00457	0.00956
NHIS NSC	0.374	0.475	0.432	0.426	0.393	0.461
Truven MDCD	-0.0772	0.639	0.904	0.96	0.88	0.998
Cerner UT	0.688	0.657	0.295	0.345	0.282	0.418

Columbia	NA	NA	NA	NA	NA	NA
Stanford	NA	NA	NA	NA	NA	NA
			Esophageal Cancer			
P-Plus	-0.769	0.316	0.0149	0.0171	0.0145	0.0204
Optum CEDM	0.114	0.366	0.755	0.734	0.703	0.766
Truven CCAE	-0.187	0.426	0.661	0.7	0.666	0.735
Truven MDCR	0.884	0.532	0.0967	0.0848	0.0792	0.0912
NHIS NSC	0.325	1.16	0.78	0.776	0.758	0.795
Truven MDCD	NA	NA	NA	NA	NA	NA
Cerner UT	NA	NA	NA	NA	NA	NA
Columbia	NA	NA	NA	NA	NA	NA
Stanford	NA	NA	NA	NA	NA	NA
			Osteonecrosis of Jaw			
P-Plus	0.968	0.772	0.21	0.207	0.198	0.217
Optum CEDM	0.607	0.63	0.335	0.327	0.315	0.339
Truven CCAE	-0.0139	0.65	0.983	0.989	0.966	0.999
Truven MDCR	0.52	1.17	0.657	0.636	0.625	0.647
NHIS NSC	NA	NA	NA	NA	NA	NA
Truven MDCD	NA	NA	NA	NA	NA	NA
Cerner UT	NA	NA	NA	NA	NA	NA
Columbia	NA	NA	NA	NA	NA	NA
Stanford	NA	NA	NA	NA	NA	NA

¶lb: lower bound
§ub: upper bound

eTable 6. Original estimates and p-values for alternative analysis, with negative control calibrated p-values. Bounds on calibrated p-values calculated from the 95% bounds of original estimate

	Original Estimate			Calibrated P-value		
Data source	Mean	SD	p-value	p-value	95% ¶lb	95% §ub
			Hip Fracture			
Optum CEDM	0.0949	0.209	0.65	0.343	0.251	0.47
Truven CCAE	-0.111	0.392	0.776	0.936	0.833	0.996
Truven MDCR	-0.243	0.146	0.0972	0.33	0.206	0.507
Truven MDCD	0.59	1.19	0.62	0.477	0.372	0.595
Columbia	NA	NA	NA	NA	NA	NA
Stanford	NA	NA	NA	NA	NA	NA
			Vertebral Fracture			
Optum CEDM	-0.143	0.168	0.395	0.891	0.717	0.994
Truven CCAE	-0.151	0.277	0.585	0.8	0.66	0.941
Truven MDCR	-0.126	0.153	0.409	0.843	0.637	0.991
Truven MDCD	-0.0233	0.838	0.978	0.779	0.592	0.965
Columbia	NA	NA	NA	NA	NA	NA
Stanford	NA	NA	NA	NA	NA	NA
			Atypical Femoral Fracture			
Optum CEDM	0.78	0.777	0.315	0.25	0.228	0.276
Truven CCAE	NA	NA	NA	NA	NA	NA
Truven MDCR	0.26	0.544	0.633	0.515	0.464	0.568
Truven MDCD	NA	NA	NA	NA	NA	NA
Columbia	NA	NA	NA	NA	NA	NA
Stanford	NA	NA	NA	NA	NA	NA
			Esophageal Cancer			
Optum CEDM	-1.16	0.941	0.216	0.269	0.249	0.291
Truven CCAE	-1.38	1.4	0.327	0.356	0.337	0.375
Truven MDCR	-0.153	1.22	0.9	0.962	0.934	0.991
Truven MDCD	NA	NA	NA	NA	NA	NA
Columbia	NA	NA	NA	NA	NA	NA
Stanford	NA	NA	NA	NA	NA	NA

			Osteonecrosis of Jaw			
Optum CEDM	NA	NA	NA	NA	NA	NA
Truven CCAE	NA	NA	NA	NA	NA	NA
Truven MDCR	NA	NA	NA	NA	NA	NA
Truven MDCD	NA	NA	NA	NA	NA	NA
Columbia	NA	NA	NA	NA	NA	NA
Stanford	NA	NA	NA	NA	NA	NA

¶lb: lower bound
§ub: upper bound

eFigure 1. A) year of and B) age at study entry, stratified by drug exposure and data source. Note patient counts are on the log-scale

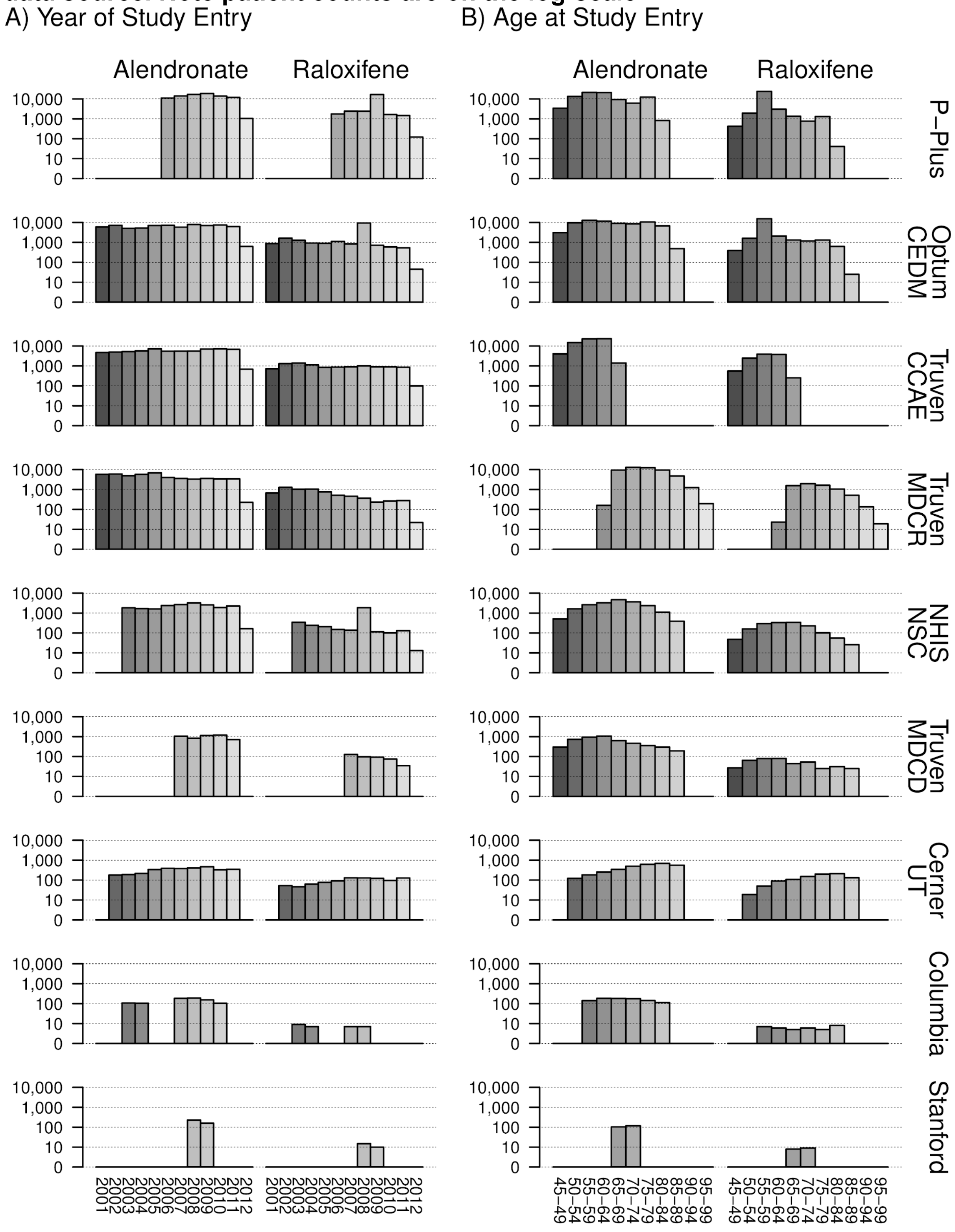

eFigure 2. Kaplan-Meier curves for Hip Fracture outcome.

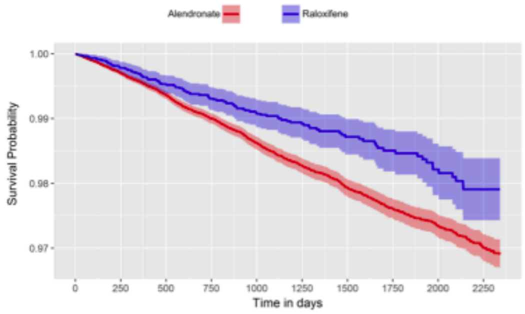

(a) P-Plus

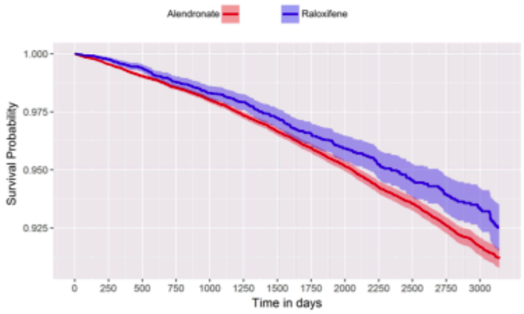

(b) Optum CEDM

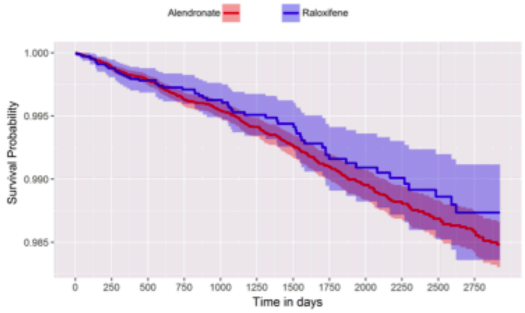

(c) Truven CCAE

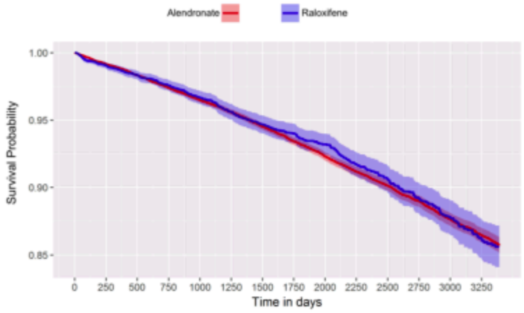

(d) Truven MDCR

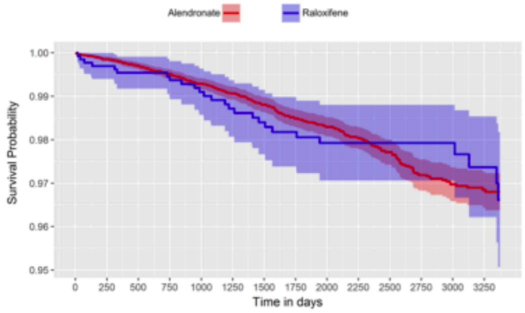

(e) NHIS NSC

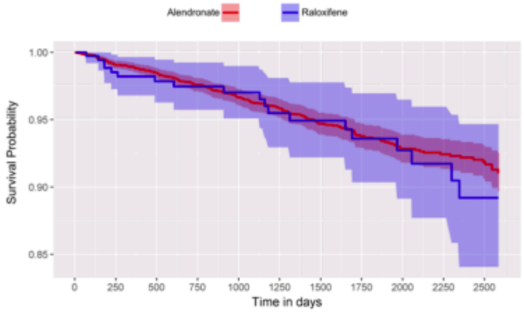

(f) Truven MDCD

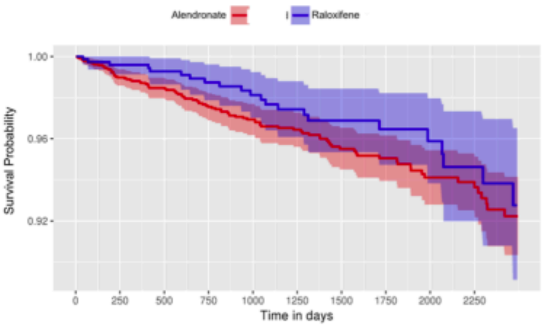

(g) Cerner UT

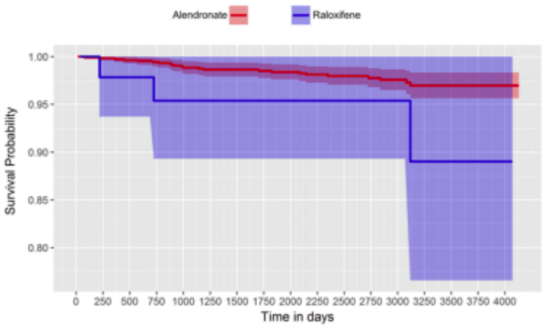

(h) Columbia

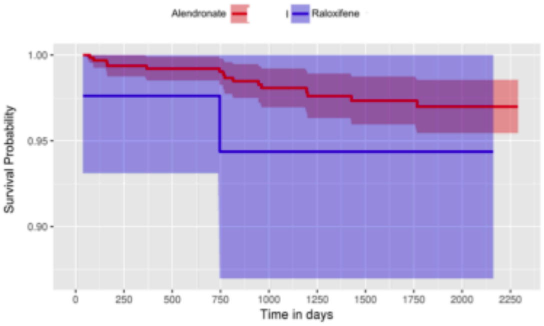

(i) Stanford

eFigure 3. Outcome Assessments of Alternative Analysis in vertebral fracture and adverse events

Vertebral Fracture

	Hazard Ratio (95% CI)
Optum CEDM	0.87 (0.63–1.22)
Truven CCAE	0.86 (0.52–1.53)
Truven MDCR	0.88 (0.66–1.20)
Truven MDCD	0.98 (0.25–6.55)
Columbia	––
Stanford	––
Summary	0.87 (0.71–1.07)

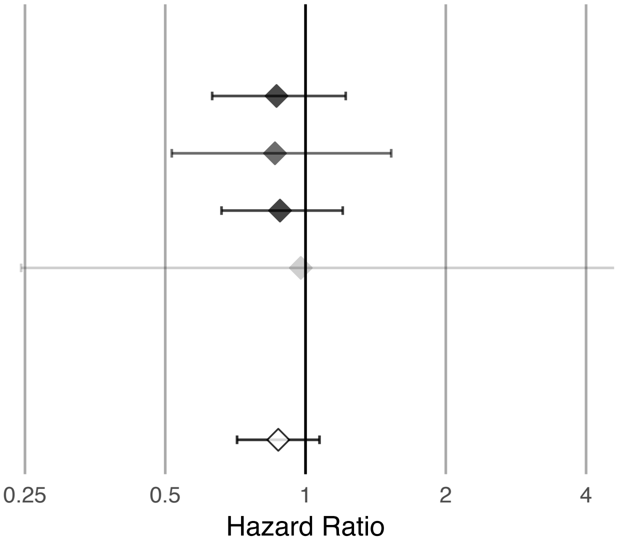

Atypical Femoral Fracture

	Hazard Ratio (95% CI)
Optum CEDM	2.18 (0.65–13.6)
Truven CCAE	––
Truven MDCR	1.30 (0.52–4.35)
Truven MDCD	––
Columbia	––
Stanford	––
Summary	1.54 (0.64–3.69)

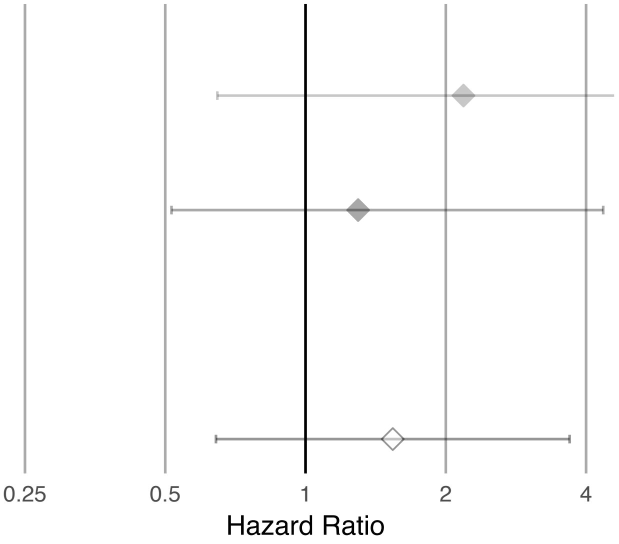

Esophageal Cancer

	Hazard Ratio (95% CI)
Optum CEDM	0.31 (0.06–2.32)
Truven CCAE	0.25 (0.02–5.59)
Truven MDCR	0.86 (0.14–16.5)
Truven MDCD	––
Columbia	––
Stanford	––
Summary	0.40 (0.11–1.45)

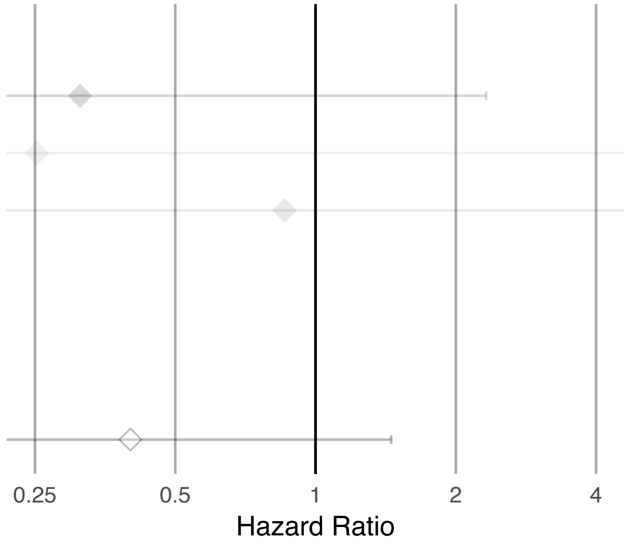

Osteonecrosis of Jaw

	Hazard Ratio (95% CI)
Optum CEDM	––
Truven CCAE	––
Truven MDCR	––
Truven MDCD	––
Columbia	––
Stanford	––
Summary	––

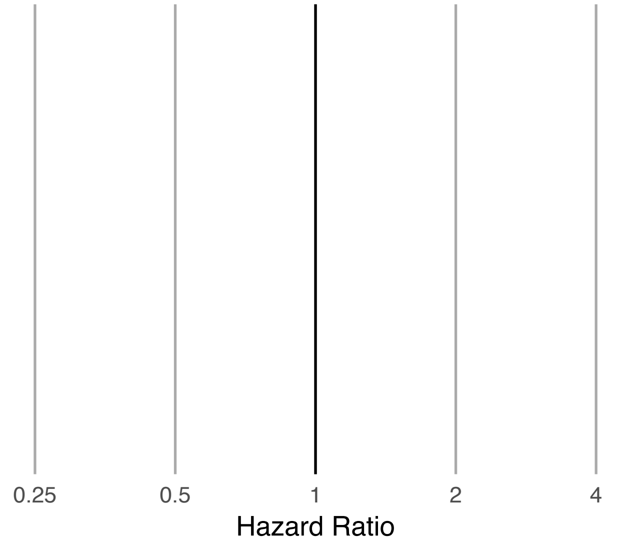

eFigure 4. Preference Score distributions in original cohort (left) and after trimming and stratification (right)

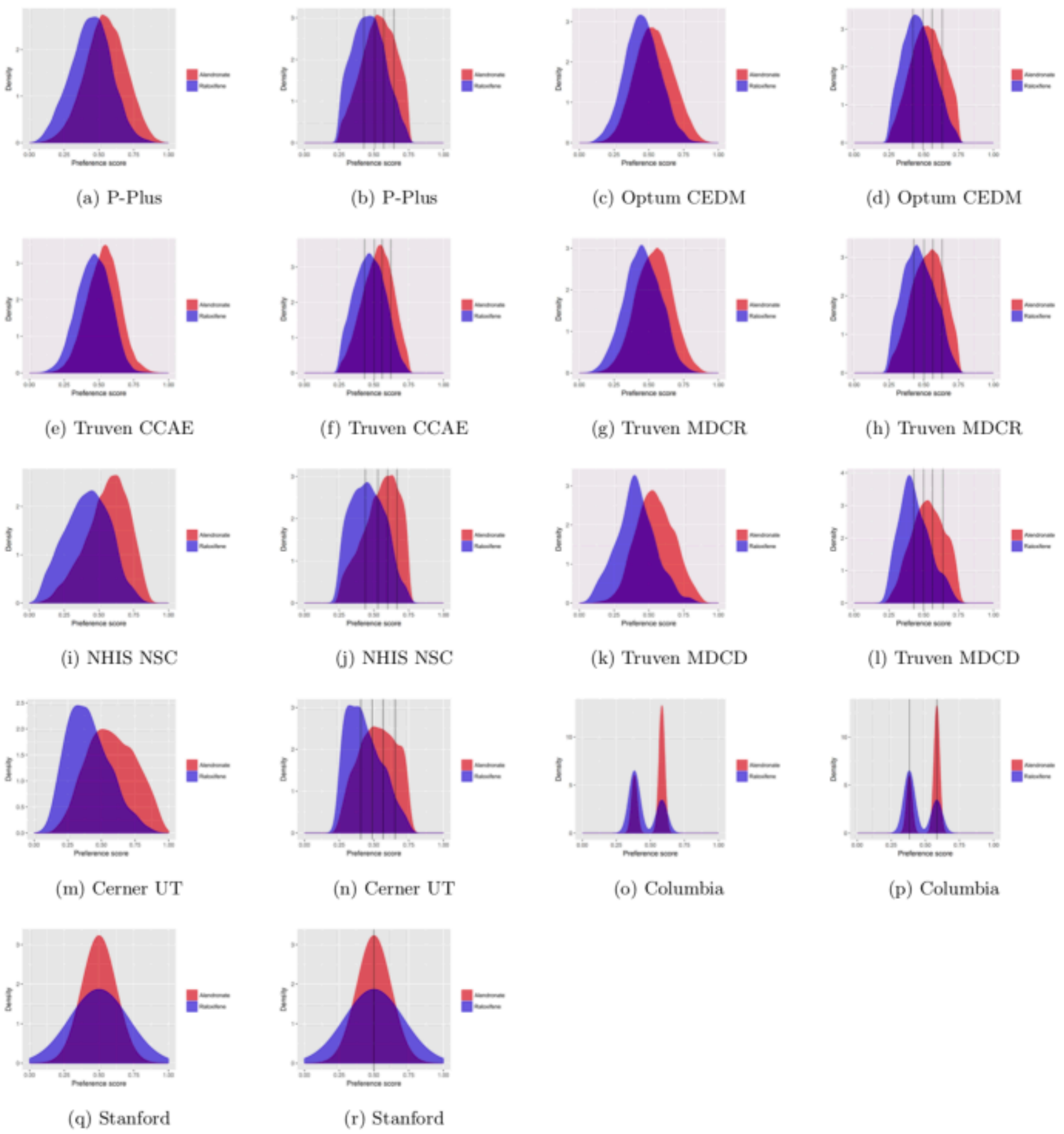

eFigure 5. Standardized difference of covariate (1 dot = 1 covariate) in each study population before and after propensity score trimming and stratification

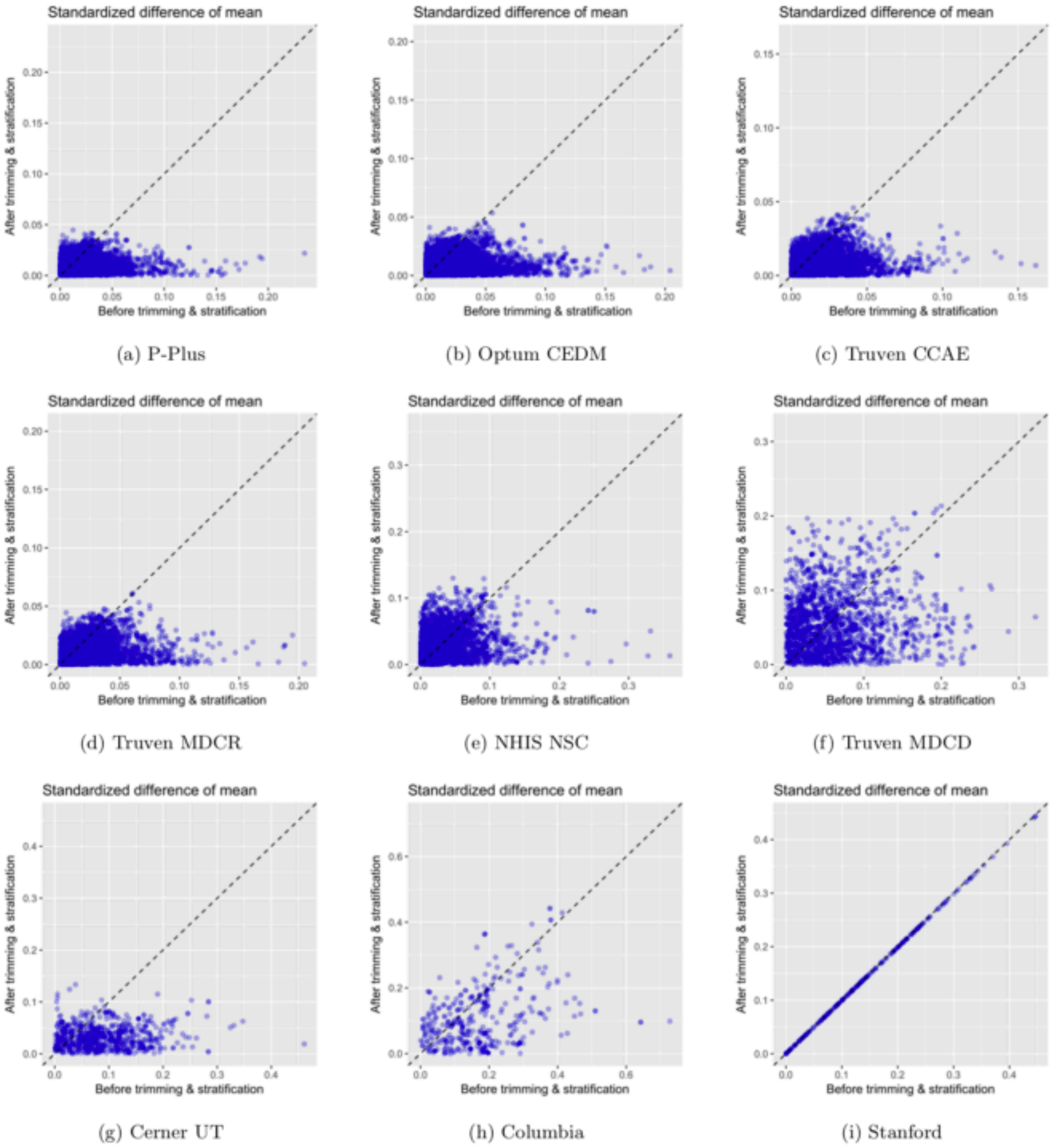

eFigure 6. P-Plus: most unbalanced covariates before (top) and after (bottom) PS trimming and stratification

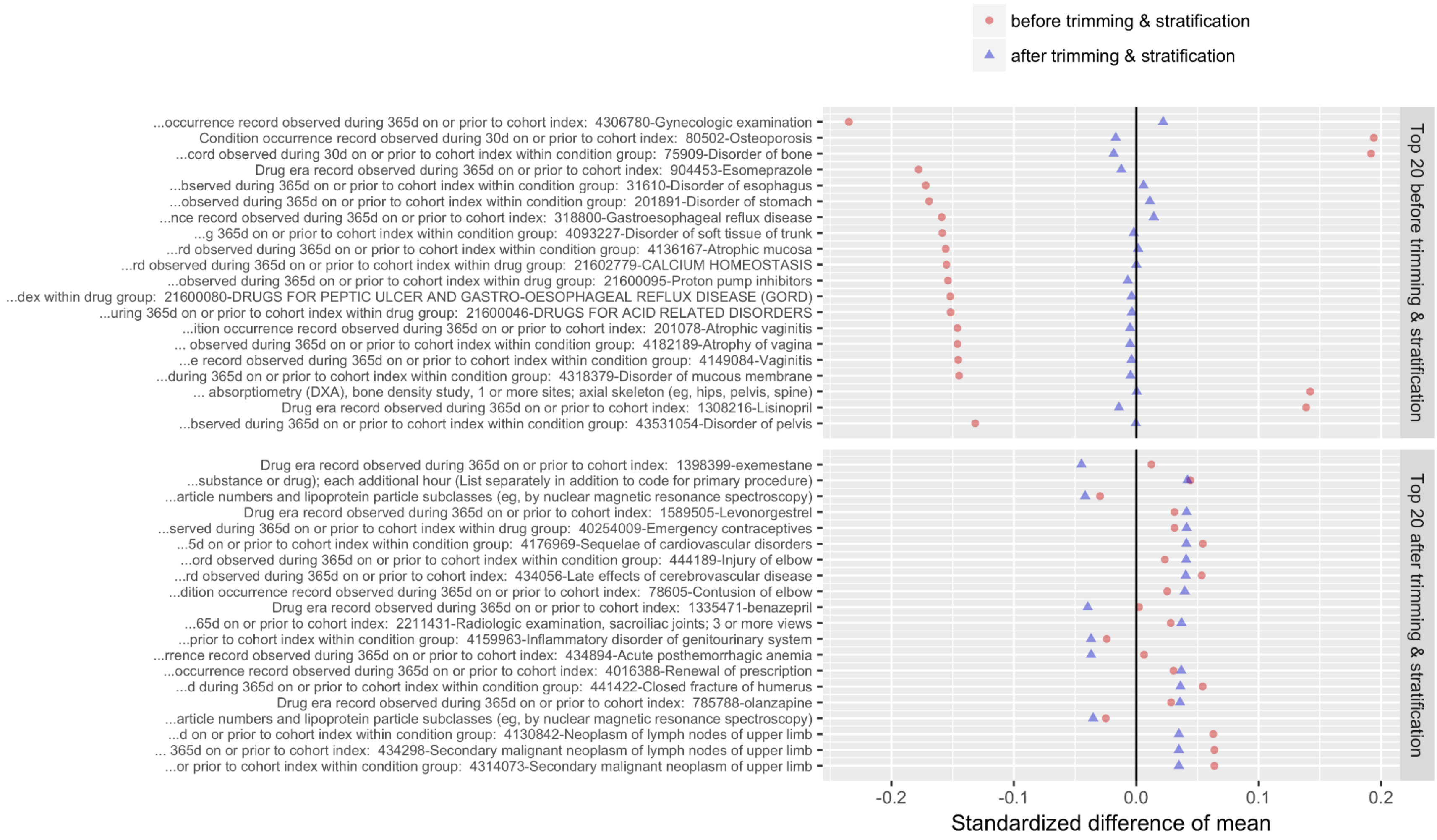

eFigure 7. Optum CEDM: most unbalanced covariates before (top) and after (bottom) PS trimming and stratification

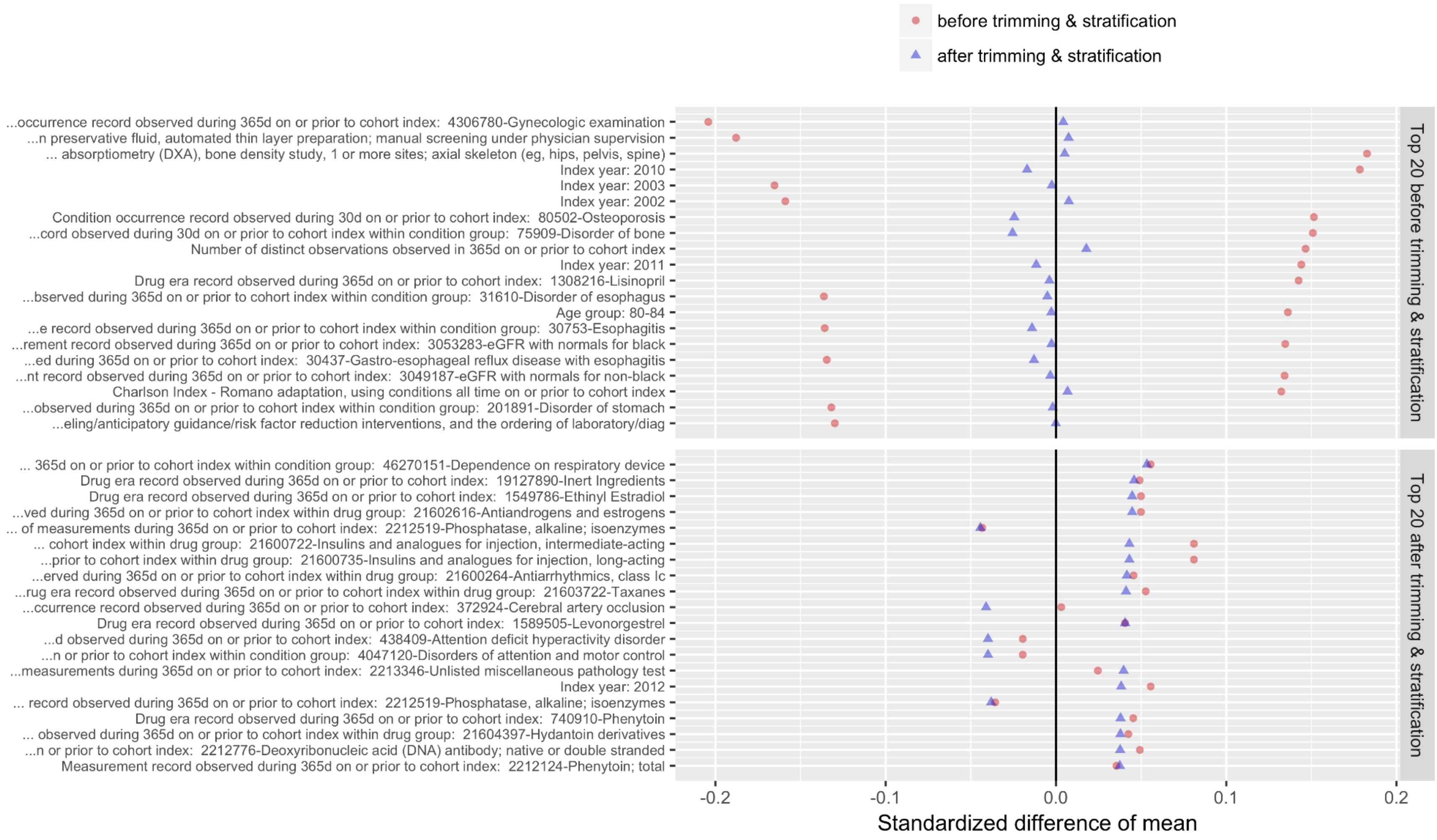

eFigure 8. Truven CCAE: most unbalanced covariates before (top) and after (bottom) PS trimming and stratification

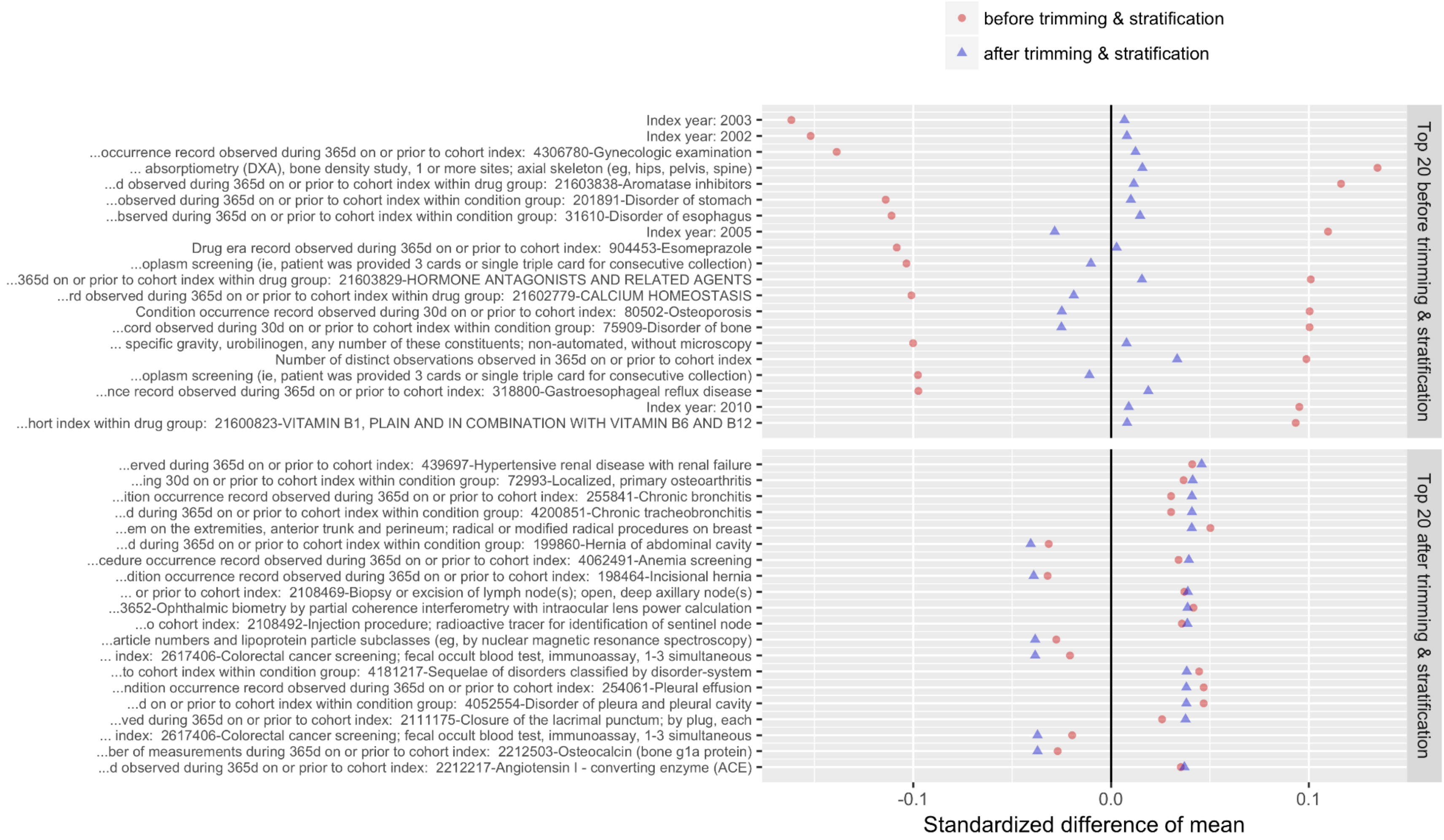

eFigure 9. Truven MDCR: most unbalanced covariates before (top) and after (bottom) PS trimming and stratification

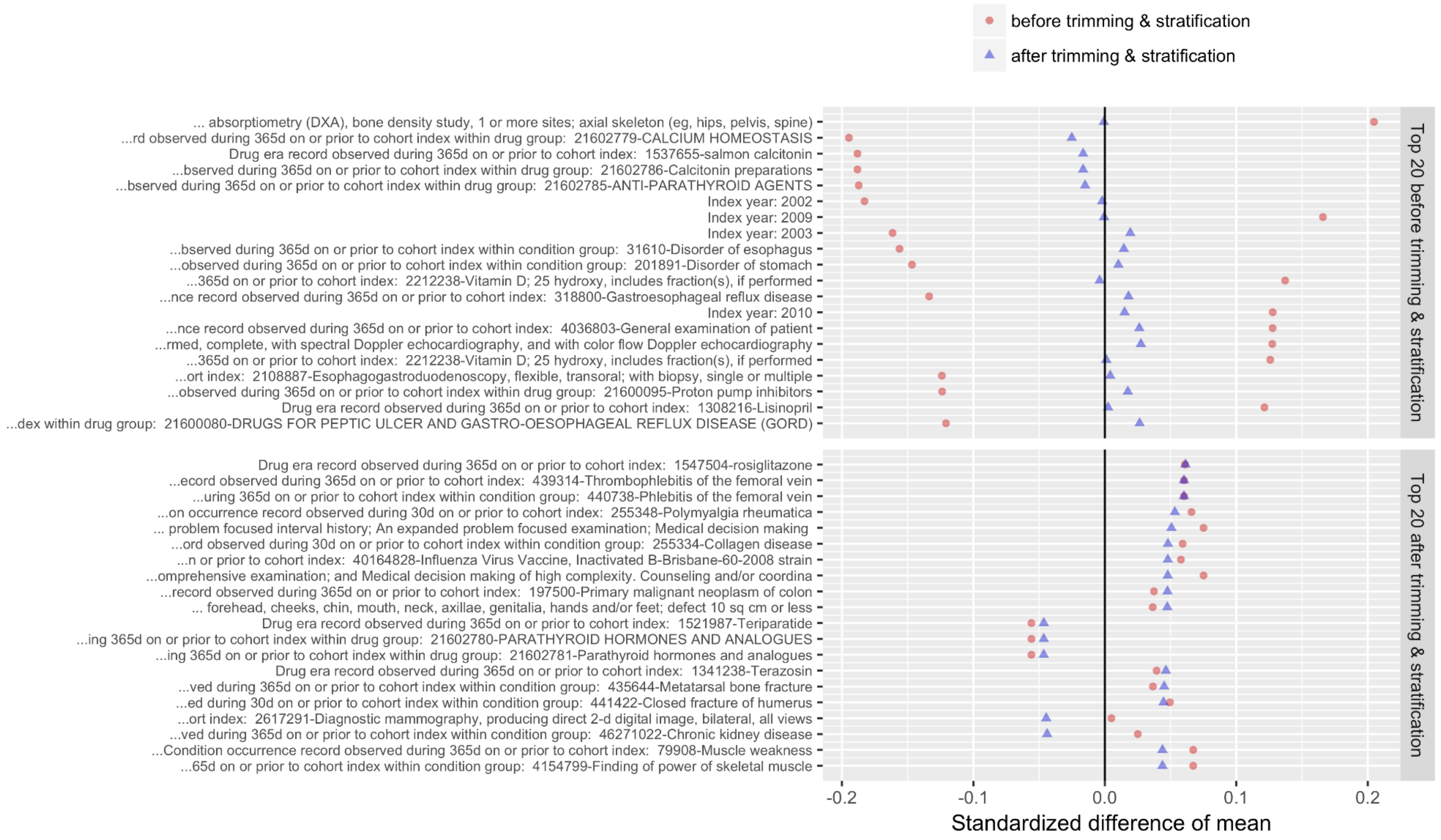

eFigure 10. NHIS NSC: most unbalanced covariates before (top) and after (bottom) PS trimming and stratification

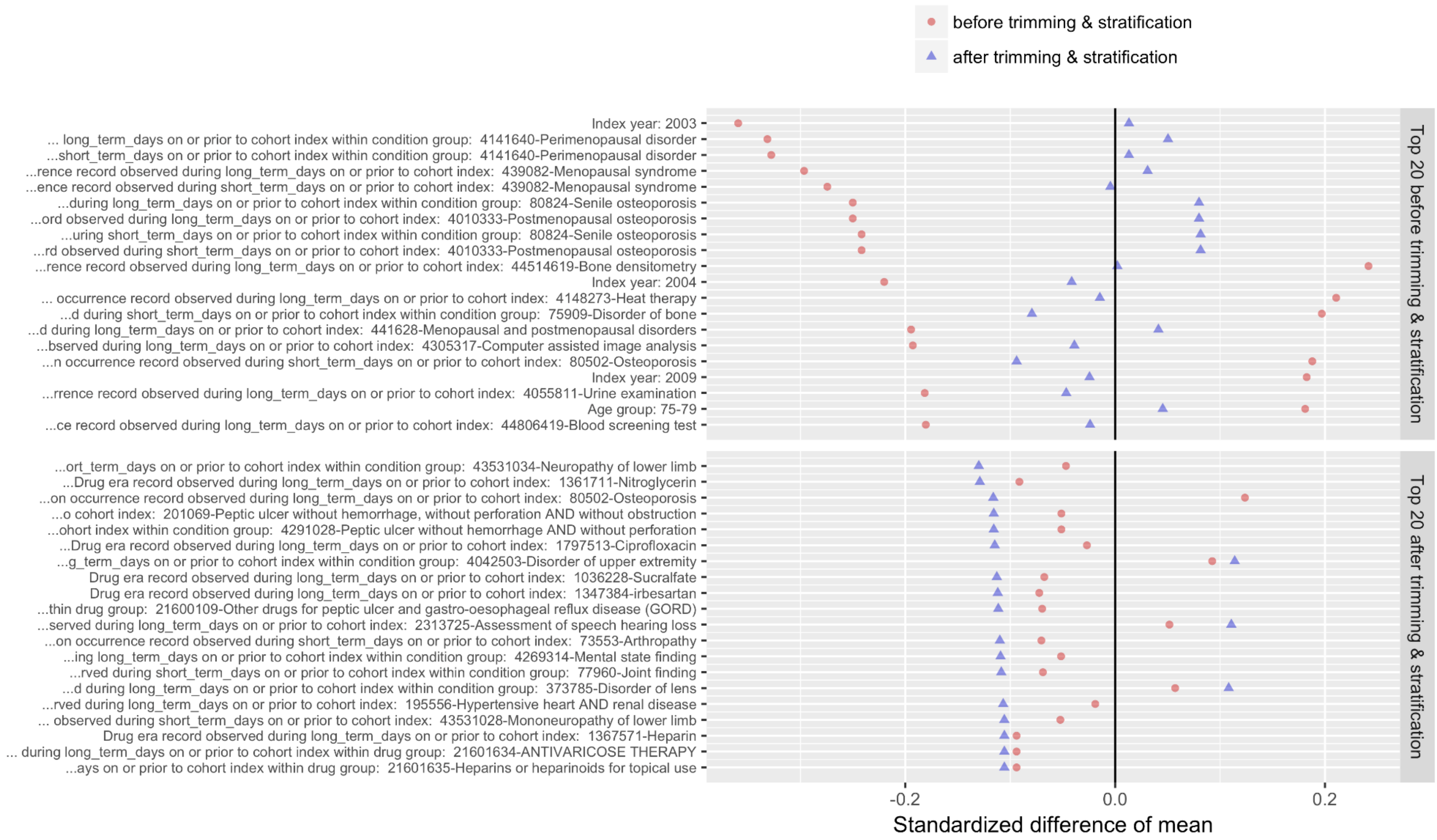

eFigure 11. Truven MDCD: most unbalanced covariates before (top) and after (bottom) PS trimming and stratification

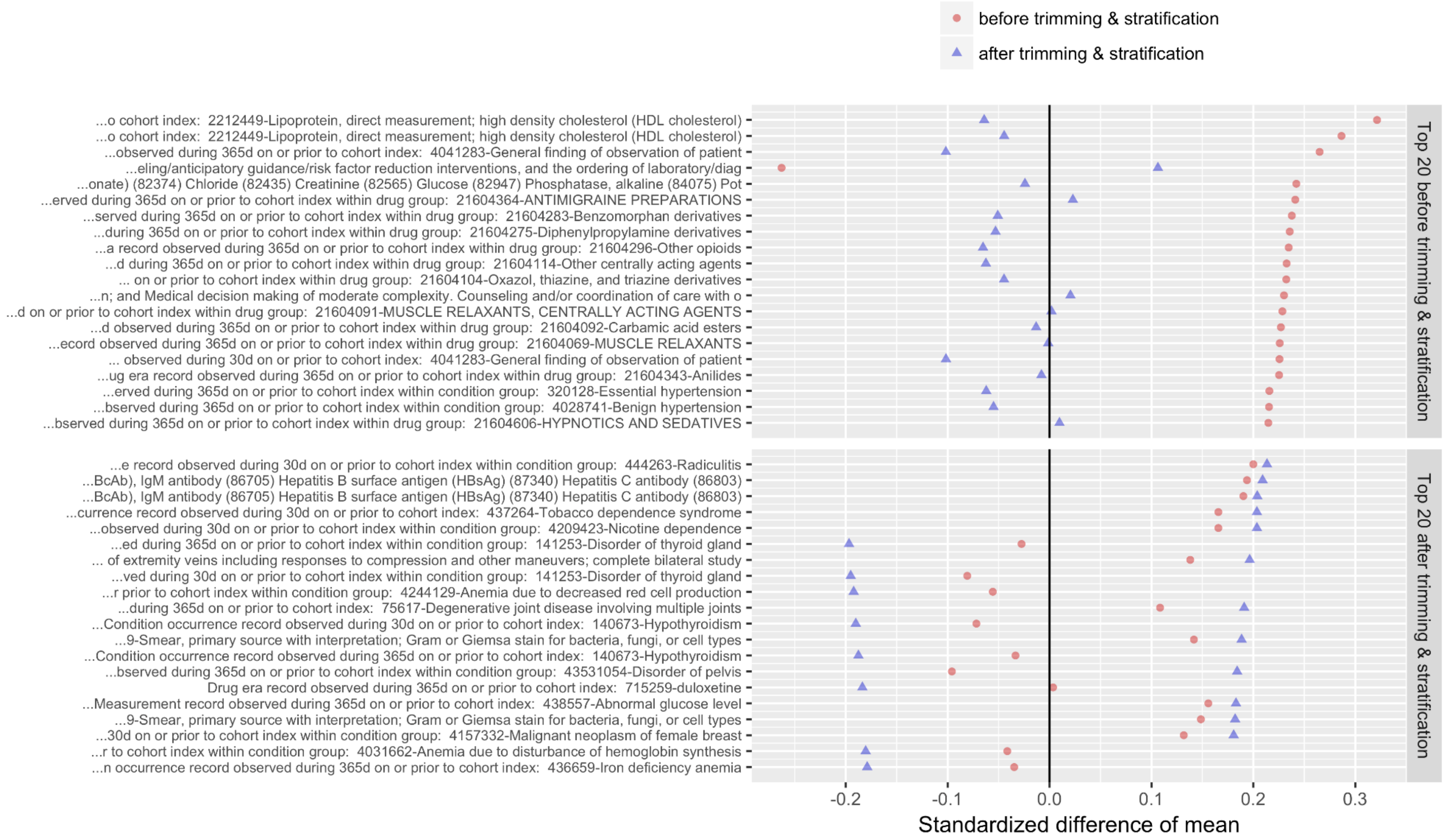

eFigure 12. Cerner UT: most unbalanced covariates before (top) and after (bottom) PS trimming and stratification

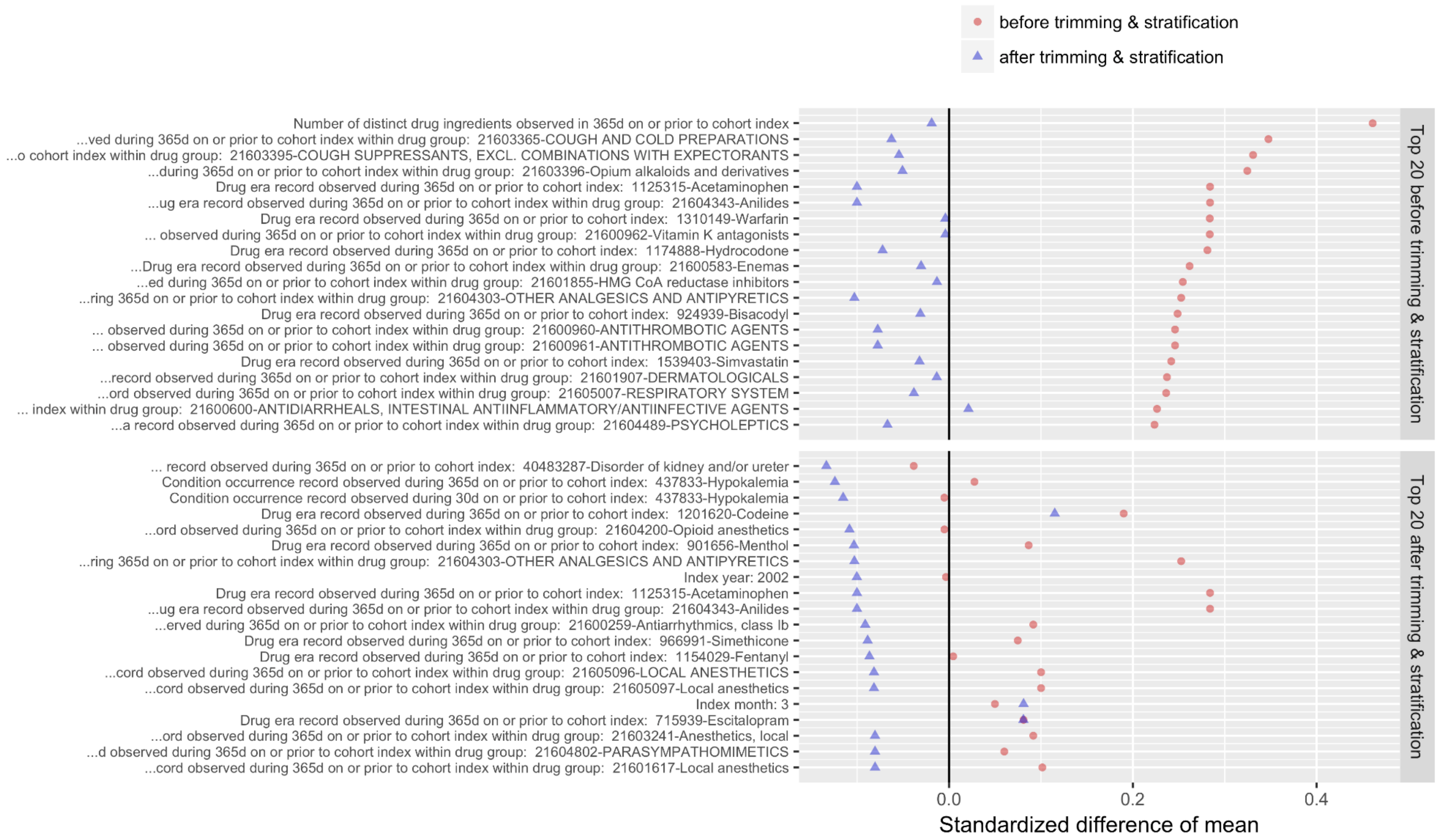

eFigure 13. Columbia: most unbalanced covariates before (top) and after (bottom) PS trimming and stratification

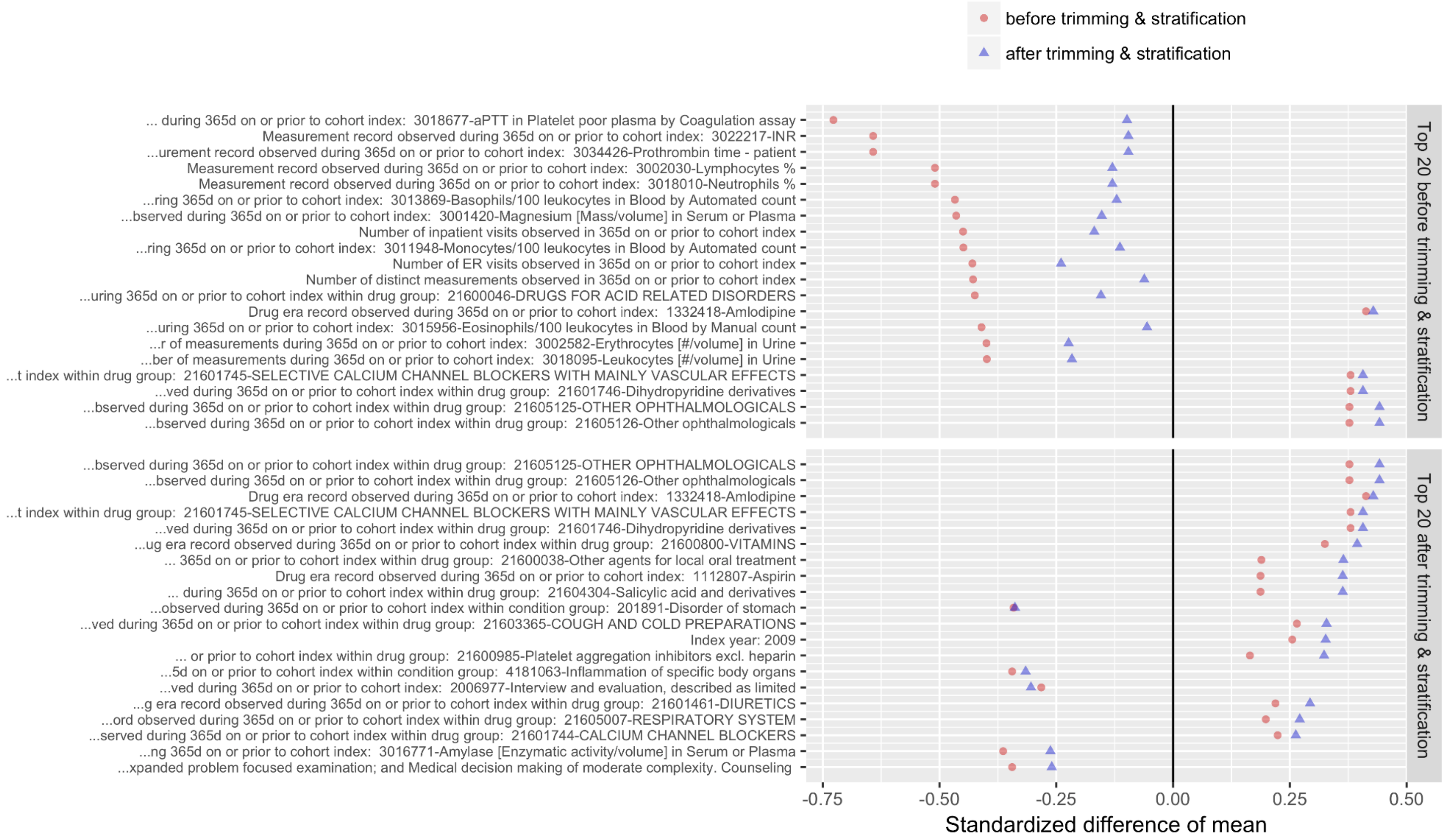

eFigure 14. Stanford: most unbalanced covariates before (top) and after (bottom) PS trimming and stratification

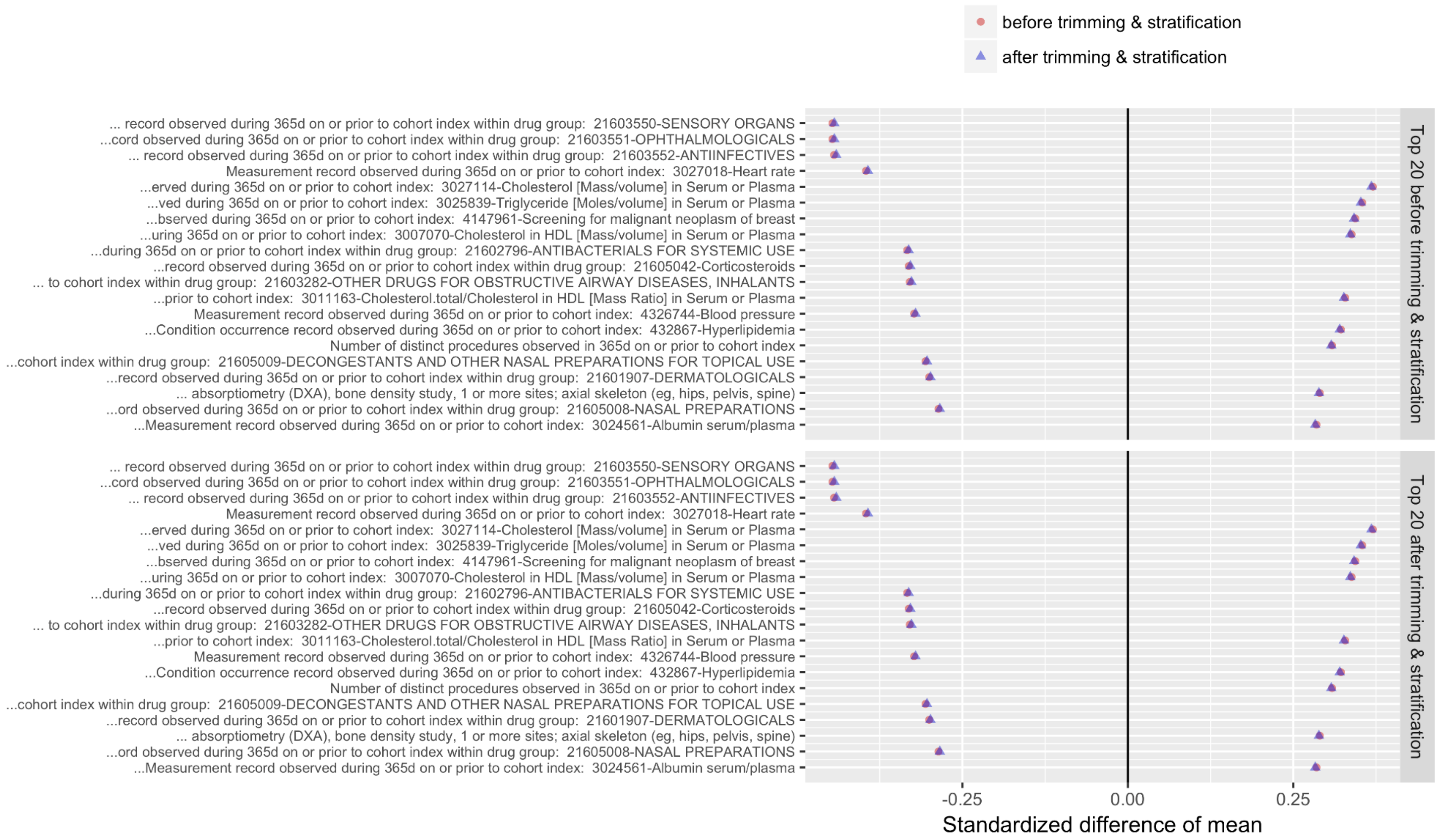

eFigure 15. Primary analysis negative control estimates (blue dots) with traditional and calibrated significance testing. Estimates below the dashed line have p<0.05 using traditional p-value calibration. Estimates in the orange areas have p<0.05 using the calibrated p-value calculation.

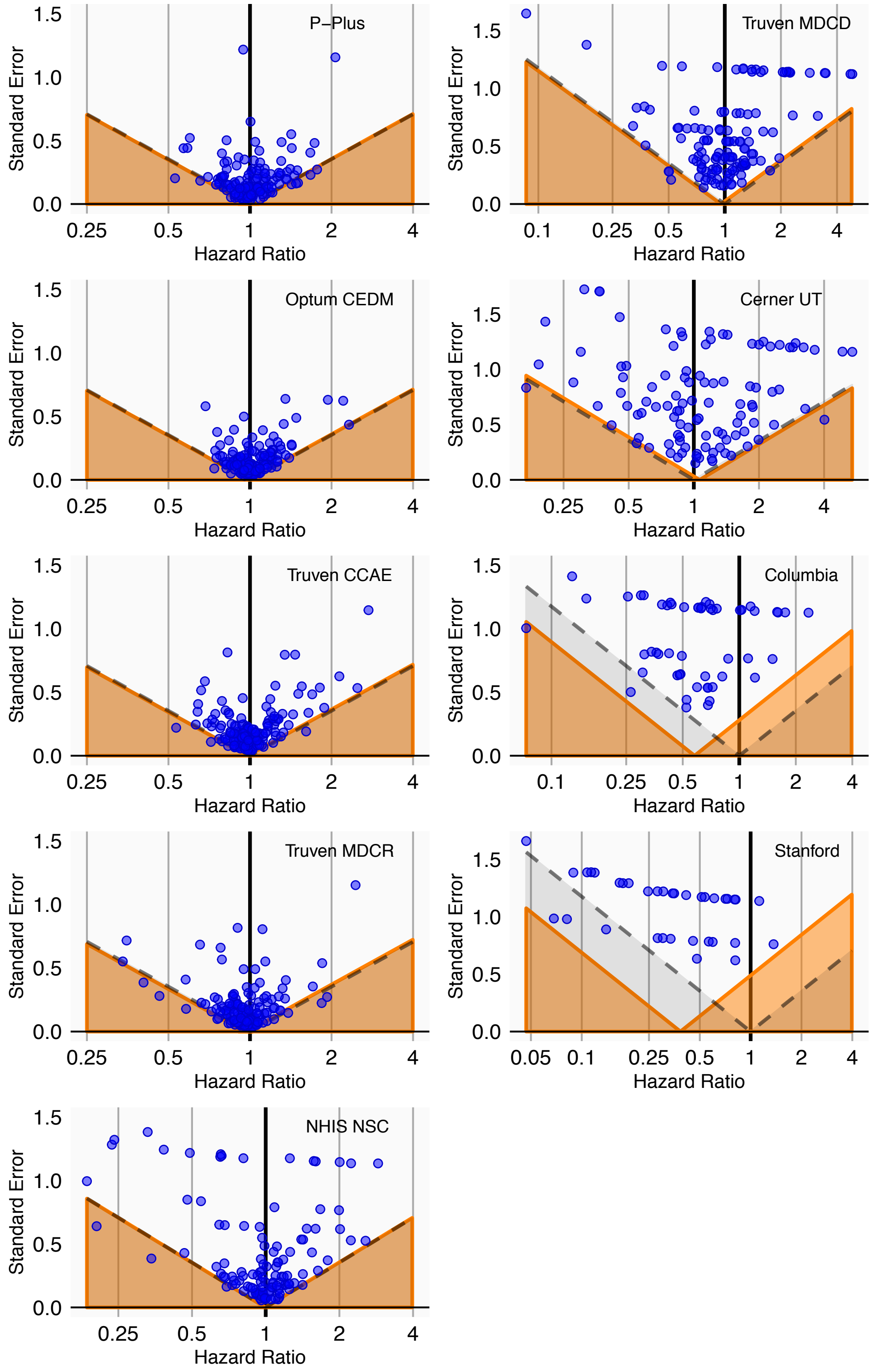

eFigure 16. Alternative analysis negative control estimates (blue dots) with traditional and calibrated significance testing. Estimates below the dashed line have p<0.05 using traditional p-value calibration. Estimates in the orange areas have p<0.05 using the calibrated p-value calculation.

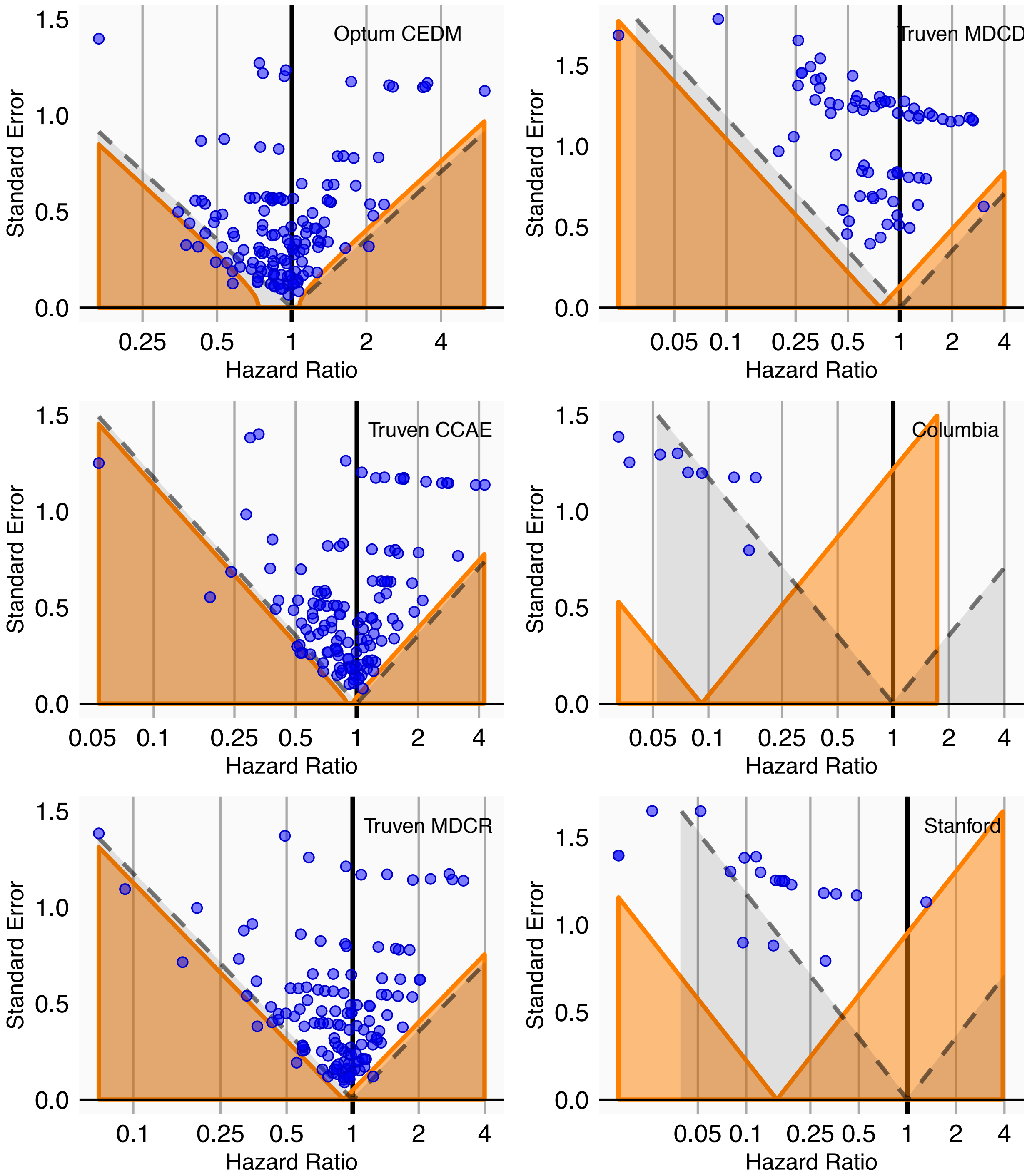

eFigure 17. Primary analysis p-value calibration plot showing the fraction of negative controls with p < α, for different levels of α. Both traditional p-value calculation and p-values using calibration are shown. For the calibrated p-value, a leave-one-out design was used.

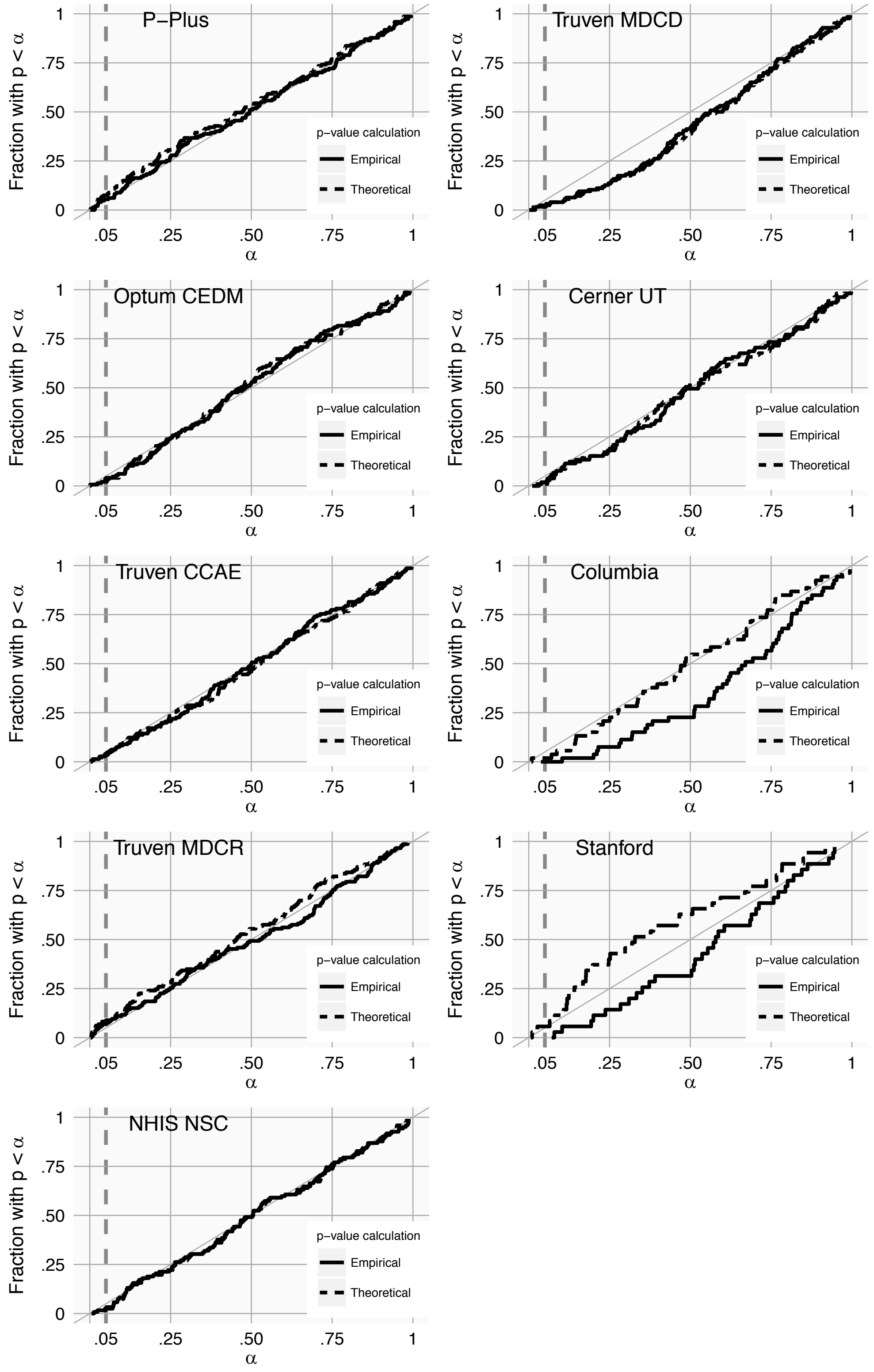

eFigure 18. Alternative analysis p-value calibration plot showing the fraction of negative controls with p < α, for different levels of α. Both traditional p-value calculation and p-values using calibration are shown. For the calibrated p-value, a leave-one-out design was used.

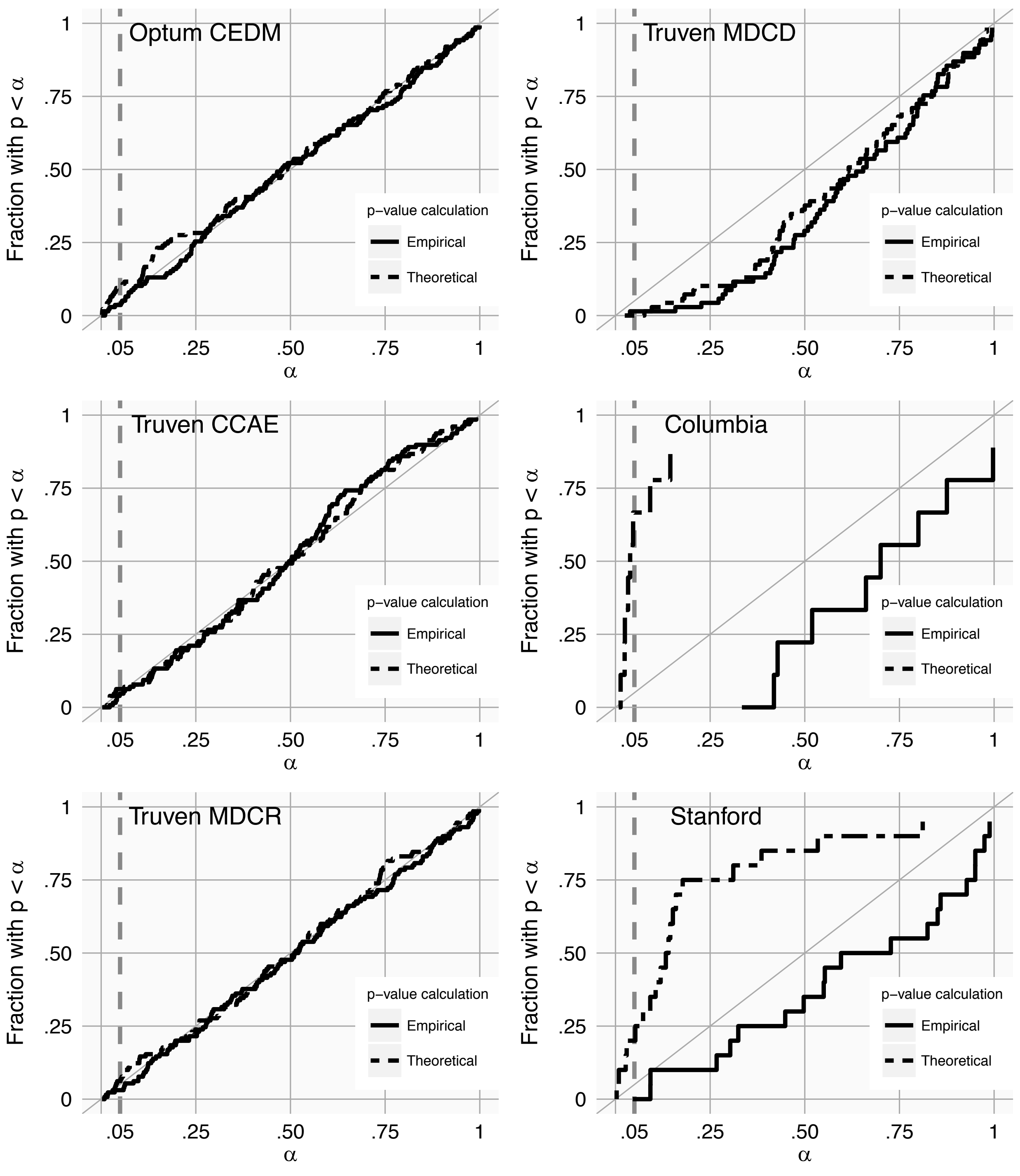

Supplement: Supplementary file 1 — Supplementary Information 1 [file 41598_2020_68037_MOESM1_ESM.pdf]
